# Kaposi’s sarcoma-associated herpesvirus viral protein kinase augments cell survival

**DOI:** 10.1038/s41419-023-06193-1

**Published:** 2023-10-18

**Authors:** Xin-Jun Wu, Zhigang Zhang, Jason P. Wong, Ricardo Rivera-Soto, Maria C. White, Aryan A. Rai, Blossom Damania

**Affiliations:** 1 Department of Microbiology and Immunology and Lineberger Comprehensive Cancer Center, the University of North Carolina at Chapel Hill, Chapel Hill, NC USA; 2 Curriculum in Genetics and Molecular Biology, the University of North Carolina at Chapel Hill, Chapel Hill, NC USA

**Keywords:** Tumour virus infections, Apoptosis

## Abstract

Oncogenic viruses have developed various strategies to antagonize cell death and maintain lifelong persistence in their host, a relationship that may contribute to cancer development. Understanding how viruses inhibit cell death is essential for understanding viral oncogenesis. Kaposi’s sarcoma-associated herpesvirus (KSHV) is associated with three different cancers in the human population, including Kaposi’s sarcoma (KS), the most common cancer in HIV patients. Previous studies have indicated that the KSHV-encoded viral protein kinase (vPK) impacts many processes dysregulated in tumorigenesis. Here, we report that vPK protects cells from apoptosis mediated by Caspase-3. Human umbilical vein endothelial cells (HUVECs) expressing vPK (HUVEC-vPK) have a survival advantage over control HUVEC under conditions of extrinsic- and intrinsic-mediated apoptosis. Abolishing the catalytic activity of vPK attenuated this survival advantage. We found that KSHV vPK-expressing HUVECs exhibited increased activation of cellular AKT kinase, a cell survival kinase, compared to control cells without vPK. In addition, we report that vPK directly binds the pleckstrin homology (PH) domain of AKT1 but not AKT2 or AKT3. Treatment of HUVEC-vPK cells with a pan-AKT inhibitor Miransertib (ARQ 092) reduced the overall phosphorylation of AKT, resulting in the cleavage of Caspase-3 and the induction of apoptosis. Furthermore, vPK expression activated VEGF/VEGFR2 in HUVECs and promoted angiogenesis through the AKT pathway. vPK expression also inhibited the cytotoxicity of cisplatin in vitro and in vivo. Collectively, our findings demonstrate that vPK’s ability to augment cell survival and promote angiogenesis is critically dependent on AKT signaling, which is relevant for future therapies for treating KSHV-associated cancers.

## Introduction

Apoptosis is regulated by extrinsic and intrinsic pathways. The extrinsic pathway can be induced through death receptor activation, cytokine, or growth factor withdrawal; intrinsic apoptosis is triggered by cellular stresses, such as DNA damage or metabolic stress [1]. Cell apoptosis is strictly controlled. AKT (also known as protein kinase B) plays an important role in the regulation of cell survival, proliferation, metabolism, and angiogenesis and is deregulated in many cancers [2, 3].

Viral proteins can inhibit cell death pathways and promote cell survival and viral-mediated oncogenesis. KSHV is one of seven human oncogenic viruses [4]. KSHV is associated with multiple cancers including Kaposi Sarcoma (KS), a tumor of endothelial origin and two types of B lymphoproliferative disorders, primary effusion lymphoma (PEL) and multicentric Castleman’s disease (MCD). KS is the most common cancer and leading cause of mortality in HIV patients [5]. Although antiretroviral therapy has reduced the incidence of KS and other HIV-related cancers [6–9], persons with HIV (PWH) who maintian undetectable plasma HIV RNA levels and a restored immune system still have a 35-fold higher risk of KS compared to the general population [8]. Additionally, KS remains the predominant cancer among PWH [7, 8].

KSHV has been shown to inhibit endothelial cell apoptosis, contributing to KS development [10]. Open reading frame (ORF) 36 of KSHV encodes a viral protein kinase (vPK), which we have previously demonstrated promotes lymphomagenesis in aged-transgenic mice [11]. We have also shown that vPK mimics the S6 kinase to promote protein synthesis and angiogenesis [12]. Fifty-nine percent of KS biopsies have detectable vPK mRNA expression [13] and Uldrick et al. showed that ORF36/vPK is a target for multicentric Castleman’s disease [14]. Thus, vPK is an attractive target for treating KSHV-associated diseases. However, the involvement of vPK in apoptotic signaling is largely unknown. We report that vPK inhibits cell apoptosis in response to cellular stresses by activating AKT1. These findings reveal that vPK may contribute to the development of KS and may represent a promising therapeutic target.

## Results

### Endothelial cells expressing vPK have increased survival upon serum deprivation

KS is an angioproliferative cancer primarily driven by endothelial cells. To identify the role of KSHV vPK in KS tumorigenesis, we generated human umbilical vein endothelial cells (HUVEC) that stably express wildtype vPK (vPK (WT)), kinase dead vPK (vPK (K108A)), or empty vector (EV) control. These cells displayed similar growth rates under 10% serum conditions (Supplementary Fig. S1a). However, cells expressing vPK (WT) had significantly higher levels of survival over time in 0% serum conditions. Moreover, mutating the vPK K108 catalytic site abolished the survival effects of vPK (Supplementary Fig. S1b).

STAT3 is involved in the regulation of cell survival, proliferation, and differentiation signals [15]. Furthermore, Punjabi et al. reported that phosphorylated active STAT3 (p-STAT3 Y705) was highly expressed in KS tumors [16]. We found that HUVEC vPK (WT) cells upregulated p-STAT3 under different serum conditions (Supplementary Fig. S1c), and p-STAT3 remained highly expressed under serum starved conditions over time (Supplementary Fig. S1d).

**Fig. 1:**
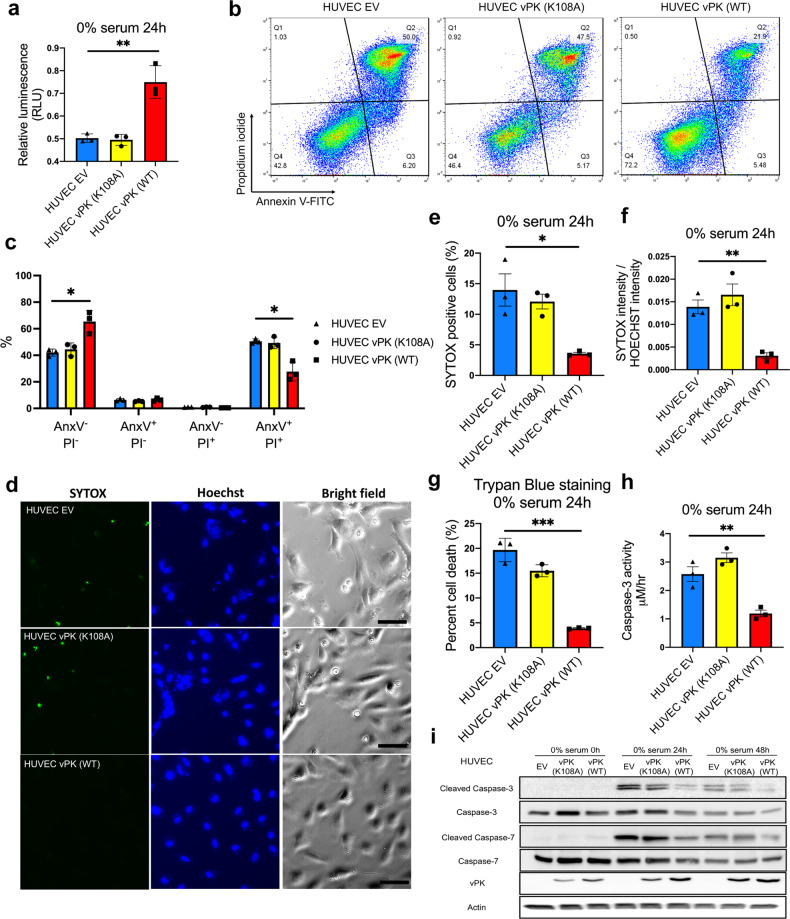
Anti-apoptotic effects of KSHV-vPK (WT) induced by serum deprivation. Viability assays were conducted with HUVEC expressing EV, vPK (K108A), and vPK (WT) cells cultured in medium without serum for 24 h. **a** Cell viability was measured by CTG assay. **b** Cells were stained with Annexin V and PI to detect the dead cells. **c** Quantitation of cells stained with Annexin V and PI. **d** Representative images of SYTOX Green and Hoechst of HUVECs treated with 0% serum medium for 24 h. The scale bar indicates 50 μm. **e** The ratio of cells stained with SYTOX Green. **f** The relative fluorescence intensity of SYTOX Green was quantitated by LAS X software. **g** Cell viability was evaluated by trypan blue staining. **h** Caspase-3 activity was measured following serum starvation for 24 h. **i** Immunoblotting demonstrating Caspase-3 and Caspase-7 cleavage levels. Actin served as a control. All data represent three repeats (*n* = 3) with three independent experiments. *P < 0.05, **P < 0.01, and ***P < 0.001.

Serum starvation primarily induces cell apoptosis by depriving cells of external survival signals including those from nutrient and growth factors. Therefore, we investigated whether HUVEC vPK (WT) inhibited apoptosis under serum deprivation. HUVEC vPK (WT) cells displayed higher viability compared with HUVEC EV and vPK (K108A) cells after serum starvation for 24 h using a CellTiter-Glo (CTG) assay (Fig. 1a). We also examined Annexin V and propidium iodide staining by flow cytometry. vPK (WT) cells exhibited approximately 50% less cell death compared to the EV control or the catalytically dead (K108A) vPK mutant under serum starvation conditions (Fig. 1b, c). Next, we investigated cell viability using SYTOX Green staining [17]. We found that HUVEC vPK (WT) cells showed less SYTOX intensity and a lower SYTOX positivity rate under serum starvation conditions for 24 h (Fig. 1d–f). Trypan blue staining confirmed that HUVEC vPK (WT) cells exhibited less cell death (Fig. 1g). Moreover, vPK (WT) expressing cells displayed significantly lower Caspase-3 activity induced by serum deprivation (Fig. 1h), in contrast to the vPK kinase-dead expressing cells. We also found higher levels of cleaved Caspase-3 and Caspase-7 activity in HUVEC EV and vPK (K108A) cells under serum starvation conditions compared to HUVEC vPK (WT) cells (Fig. 1i). These data suggest that the expression of wild-type vPK inhibits cell death induced by serum starvation.

### vPK suppresses intrinsic apoptosis

**Fig. 2:**
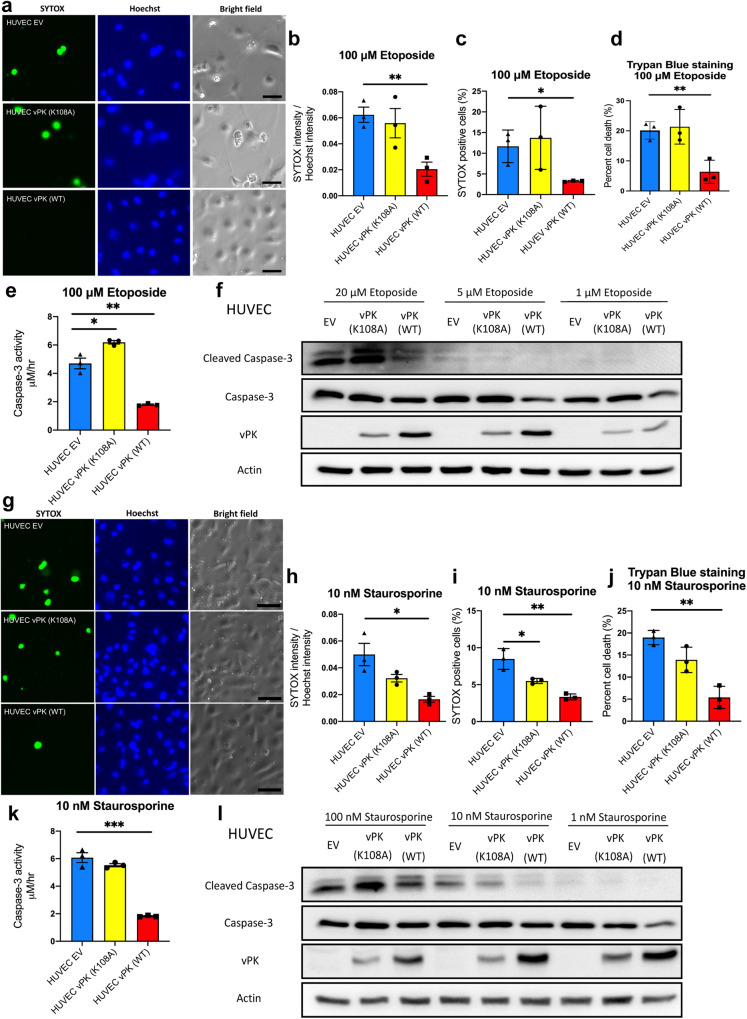
vPK expression confers endothelial cell survival upon etoposide or staurosporine treatment. HUVEC expressing EV, vPK (K108A), and vPK (WT) cells were cultured with complete medium and treated with etoposide **a**–**f** and staurosporine **g**–**l** for 24 h. **a** Representative images of SYTOX and Hoechst-positive HUVECs treated with etoposide for 24 h. The scale bar indicates 50 μm. **b** Relative fluorescence intensity of SYTOX Green after 24 h etoposide treatment. **c** The ratio of cells stained with SYTOX Green after 24 h etoposide treatment. **d** Cell viability was evaluated by trypan blue staining after etoposide treatment for 24 h. **e** Caspase-3 activities in HUVECs after being treated with etoposide for 24 h. **f** Immunoblotting revealed cleaved Caspase-3 expression after different etoposide treatments. Actin served as a control. **g** Representative images of SYTOX and Hoechst-positive HUVECs treated with staurosporine for 24 h. The scale bar indicates 50 μm. **h** The relative fluorescence intensity of SYTOX Green after 24 h staurosporine treatment. **i** The ratio of cells stained with SYTOX Green after 24 h staurosporine treatment. **j** Cell viability was evaluated by trypan blue staining after staurosporine treatment for 24 h. **k** Caspase-3 activity in HUVECs was measured after cells were treated with staurosporine for 24 h. **l** Immunoblotting demonstrates cleaved Caspase-3 expression after different staurosporine treatments. Actin served as a control. All data are representative of three biological repeats (*n* = 3) in three independent experiments. *P < 0.05, **P < 0.01, and ***P < 0.001.

To investigate whether vPK can also inhibit apoptosis induced by different intrinsic pathways, we treated HUVECs with etoposide and staurosporine. Etoposide is an inhibitor of DNA topoisomerase II resulting in DNA damage [18], while staurosporine is a relatively broad protein kinase inhibitor [19, 20]. After treatment with etoposide for 24 h, there was less SYTOX intensity and positivity in HUVEC vPK (WT) compared to the EV control and the vPK (K108A) cells (Fig. 2a–c). Trypan blue staining showed HUVEC vPK (WT) cells had less cell death when treated with etoposide for 24 h (Fig. 2d). Similarly, the activity of Caspase-3 was lower in HUVEC vPK (WT) cells (Fig. 2e), which was confirmed by decreased expression of cleaved Caspase-3 (Fig. 2f) in a dose-dependent manner.

Similarly, HUVEC vPK (WT) cells displayed less SYTOX intensity and lower SYTOX-positive cells after staurosporine treatment for 24 h compared to the EV control and vPK (K108A) cells (Fig. 2g–i). In addition, trypan blue staining showed less cell death in HUVEC vPK (WT) after treatment with staurosporine (Fig. 2j), which was concordant with less Caspase-3 activity and cleaved Caspase-3 expression (Fig. 2k, l). In contrast, HUVEC vPK (K108A) cells had higher cleaved Caspase-3 levels than HUVEC vPK (WT) (Fig. 2l). Therefore, vPK inhibits intrinsic apoptosis induced by DNA damage or metabolic stress.

### vPK-expressing endothelial cells display higher CEACAM6 expression and activated AKT

**Fig. 3:**
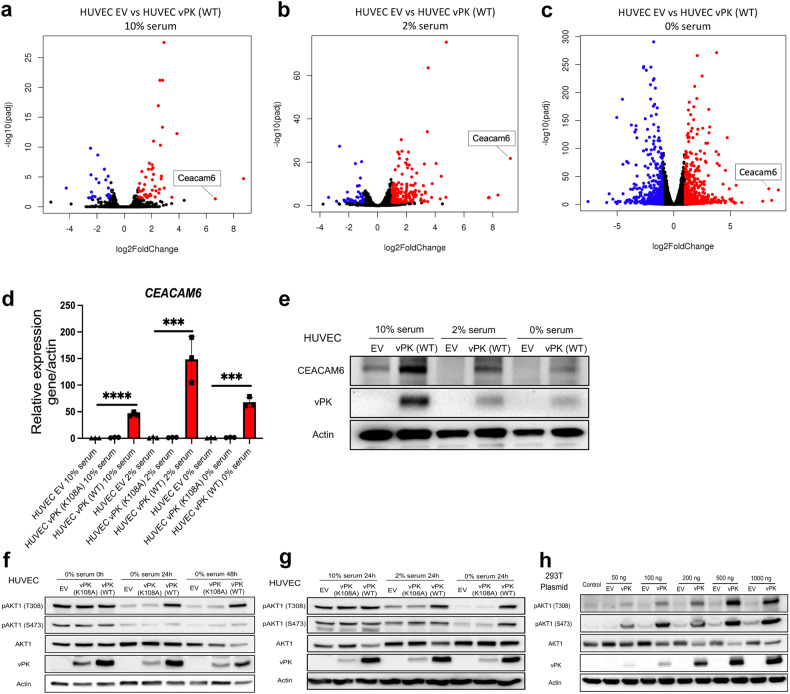
Overexpression of CEACAM6 and activation of AKT pathway in HUVEC vPK (WT) cells. **a**–**c** Volcano plots of the RNA-seq data depicting the fold change (log2FoldChange) versus the statistical significance of differential expression (-log10 (adjusted *P* value)) in three different concentrations of serum (*n* = 2). Up-regulated (red) and down-regulated (blue) genes have an adjusted *P* value < 0.05 and log2FoldChange > 1 or <-1, respectively. CEACAM6 is labeled. **d** CEACAM6 expression in HUVEC EV, HUVEC vPK (K108A), and HUVEC vPK (WT) cells under different serum conditions were verified by qRT-PCR (*n* = 3). Actin served as an endogenous control. Data show mean ± SEM, unpaired two-tailed t-test. ***P < 0.001, and ****P < 0.0001. **e** Western blot analysis of CEACAM6 and vPK. Actin served as a loading control. **f** Immunoblot of phosphorylated AKT in HUVECs stably expressing EV, vPK (K108A), and vPK (WT) in serum-free medium over time. **g** Western blot of phosphorylated AKT in HUVECs stably expressing EV, vPK (K108A), and vPK (WT) in different media conditions. **h** Immunoblot of HEK293T cells transfected with increasing amounts of plasmid. Anti-V5 immunoblot was performed to verify the expression of vPK. Actin served as a control.

To investigate the mechanism by which vPK suppresses apoptosis, we performed RNA sequencing (RNA-seq) to compare the gene expression profiles between HUVEC EV and HUVEC vPK (WT) cells under 10%, 2%, and 0% serum conditions (Fig. 3a–c). A tumor promoting protein, CEACAM6 [21], was among the top three upregulated genes in HUVEC vPK (WT) cells under different serum conditions compared to HUVEC EV cells. This finding was corroborated by qPCR and Western blot (Fig. 3d, e).

Previous studies have demonstrated that CEACAM6 activates the AKT pathway [22, 23], which is known to inhibit apoptosis. Hence, we investigated whether AKT was activated in HUVEC vPK (WT) cells. vPK expression in HUVECs increased both the phosphorylation of AKT1 at Thr308 and Ser473, even under serum deprivation conditions (Fig. 3f, g). Phosphorylation on either Thr308 or Ser473 activates AKT, and this phosphorylated form of AKT (pAKT) has been associated with inhibiting apoptosis, and inducing proliferation and cell motility [24]. Interestingly, HUVEC vPK (K108A) cells had similar AKT phosphorylation levels compared to HUVEC EV under different serum conditions (Fig. 3f, g). To confirm wild-type vPK can activate AKT1, plasmids expressing vPK or an empty control were transfected into HEK293T cells for 24 h. Levels of pAKT (T308) and pAKT (S473) were higher in vPK (WT)-expressing cells compared with empty vector control cells. Transfection of increasing amounts of WT vPK expression plasmid resulted in a dose-dependent increase of AKT phosphorylation at both residues T308 and S473 (Fig. 3h).

To confirm these results, pooled HUVECs expressing empty vector, wild-type vPK, or mutant vPK were separated and underwent single colony selection (Supplementary Fig. S2). Levels of pAKT (T308 and S473) were significantly higher in vPK (WT)-expressing HUVECs compared to empty vector control and vPK (K108A) cells under serum starvation (Supplementary Fig. S2). These results indicate that higher levels of active phosphorylated AKT in HUVEC vPK (WT) cells are dependent on the kinase activity of vPK.

### vPK interacts with AKT1, but not AKT2 or AKT3

**Fig. 4:**
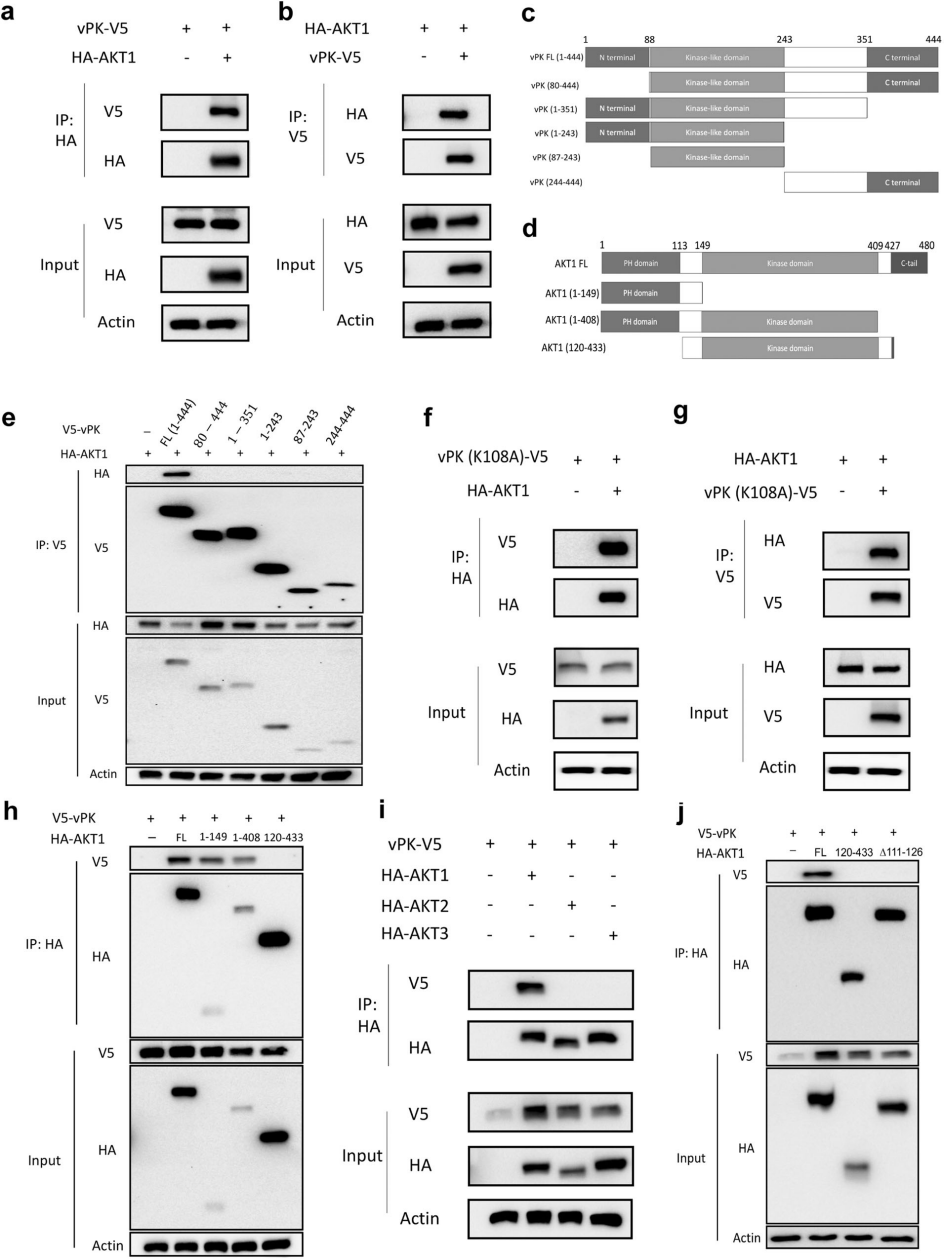
vPK interacts with AKT1 through its PH domain. **a**, **b** HA-AKT1 and V5-vPK expressing plasmids were transfected into HEK293T cells individually or together. Cell lysates were immunoprecipitated with anti-HA (**a**) or anti-V5 (**b**) beads overnight and Western blotting was performed with anti-V5 or anti-HA antibodies. **c** Schematic representation of wildtype vPK and its deletion constructs. **d** Schematic representation of wildtype AKT1 and its deletion constructs. **e** HA-tagged AKT1 and V5-tagged vPK mutations were transfected individually or together into HEK293T cells. Anti-V5 beads were used to immunoprecipitate cell lysates and then Western blotting was performed with the indicated antibodies. **f**, **g** HA-tagged AKT1 and V5-tagged vPK (K108A) plasmids were transfected into HEK293T cells individually or together for 30 h. Cell lysates were incubated with anti-HA (**f**) or anti-V5 (**g**) beads overnight and immunoblotted with the indicated antibodies. **h** HA-tagged AKT1 mutants and V5-tagged vPK were transfected individually or together into HEK293T cells for 30 h. Anti-HA beads were used to immunoprecipitate cell lysates and Western blotting with the indicated antibodies. **i** HA-tagged AKT1, AKT2, AKT3, and V5-tagged vPK plasmids were transfected individually or together into HEK293T cells. Anti-HA beads were used to immunoprecipitate cell lysates and Western blotting was performed with the indicated antibodies. **j** V5-vPK failed to pull down HA-AKT1 lacking amino acids from 111 to 126.

To determine how vPK activates AKT, we investigated whether vPK interacts with AKT1. We expressed vPK-V5 and AKT1-HA-tagged proteins in HEK293T cells. We chose the V5 tag because of its small size and versatility. We used HA-antibody-conjugated beads to pull down AKT1 and found that vPK coimmunoprecipitated with AKT1 (Fig. 4a). We also performed the reverse IP by performing coimmunoprecipitations with V5-antibody-conjugated beads to immunoprecipitate V5-tagged vPK (WT) and found vPK coimmunoprecipitated with AKT1 (Fig. 4b). Interestingly, BGLF4, the Epstein-Barr virus homolog of KSHV vPK, also binds to AKT1 (Supplementary Fig. S3).

We examined the domains of interaction between vPK and AKT1 (Fig. 4c, d). vPK has a N-terminal domain, a kinase-like domain, and a C-terminal domain. We generated 5 truncated forms of vPK-V5; however, all of these truncated forms of vPK-V5 could not bind AKT1-HA (Fig. 4e), indicating the entire length of vPK is required to form a physical complex with AKT1. Furthermore, the kinase-dead form of vPK can still immunoprecipitate with HA-AKT1 (Fig. 4f, g). To investigate which domain of AKT1 is essential for the interaction with vPK, we performed co-IPs of vPK-V5 with different AKT1 mutant plasmids. AKT1 has three domains: the N-terminal PH domain is important for the autoinhibition of AKT activity, a central kinase domain, and a C-terminal regulatory domain. We found that AKT1 lacking the PH domain did not interact with vPK (Fig. 4h), indicating that vPK binds the PH domain of AKT1.

To our surprise, vPK only binds to AKT1 but not AKT2 or AKT3 (Fig. 4i). Since vPK binds to the PH domain, and the amino acids 111-126 of AKT1, AKT2, and AKT3 are different (Supplementary Fig. S4), we suspected that amino acids 111–126 of AKT1 are essential for the interaction between vPK and AKT1. Co-immunoprecipitation revealed that AKT1Δ111–126 -HA does not interact with vPK-V5 (Fig. 4j). These data corroborate the fact that vPK interacts with AKT1 through its PH domain.

### AKT is essential for the protective effect of vPK

**Fig. 5:**
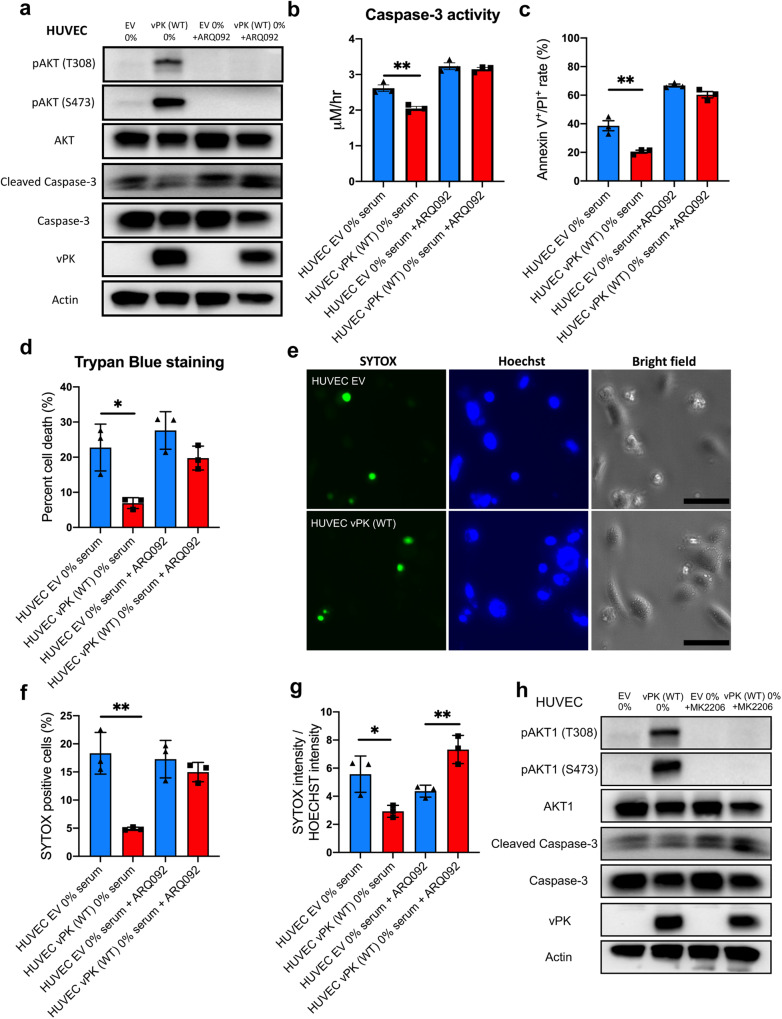
AKT1 inhibitors abolish vPK-mediated cell survival. HUVEC EV and HUVEC vPK (WT) were treated with the AKT1 inhibitor, ARQ 092 (0 or 1 μM) for 24 h in no serum conditions. **a** Western blotting showed p-AKT1 (T308), and p-AKT1 (S473) and cleaved Caspase-3 after AKT inhibitor ARQ 092 treatment. **b** Caspase-3 activity was determined by colorimetric assay. **c** Samples were stained with Annexin V and PI and analyzed using FACS. Quantitation of cells stained with Annexin V^+^/PI^+^. **d** Cell viability was evaluated by trypan blue staining. **e** Representative images of SYTOX Green and Hoechst staining of HUVECs treated with 0% serum medium with 1 μM ARQ 092 for 24 h. The scale bar indicates 50 μm. **f** Ratio of cells stained with SYTOX Green. **g** Relative fluorescence intensity of SYTOX Green shown in panel **e** was quantitated by LAS X software. **h** Cells were cultured in serum-free medium and cultured with or without MK2206 (2 μM) for 24 h. Immunoblotting was performed for the indicated proteins using the cell lysates.

To determine how AKT activation impacts cell survival, we treated HUVECs with a pan-AKT inhibitor ARQ 092. As expected, we found a significant decrease in the levels of both phosphorated AKT (T308 and S473) in HUVEC vPK (WT) cells after 24 h of treatment (Fig. 5a). This decrease in p-AKT levels also coincided with an increase in cleaved Caspase-3 expression (Fig. 5a) and activity (Fig. 5b) to levels similar to the EV control cells. Inhibition of AKT led to a significant increase in cell death under serum starvation indicating that AKT1 signaling plays a critical role in vPK’s ability to promote survival (Fig. 5c). AKT inhibition resulted in similar death rates between HUVEC EV and HUVEC vPK (WT) cells in serum-starved conditions using trypan blue staining (Fig. 5d). SYTOX staining showed similar SYTOX intensity and positivity rate between HUVEC EV and HUVEC vPK (WT) groups after ARQ 092 treatment (Fig. 5e–g). Consistent with these observations, treatment with another AKT inhibitor MK2206 also increased cell death in HUVEC vPK (WT) cells, indicated by an increase in cleaved Caspase-3 levels (Fig. 5h). Taken together, these findings demonstrate that the protective effect of wild-type vPK relies on its activation of AKT1.

### vPK promotes angiogenesis through AKT

**Fig. 6:**
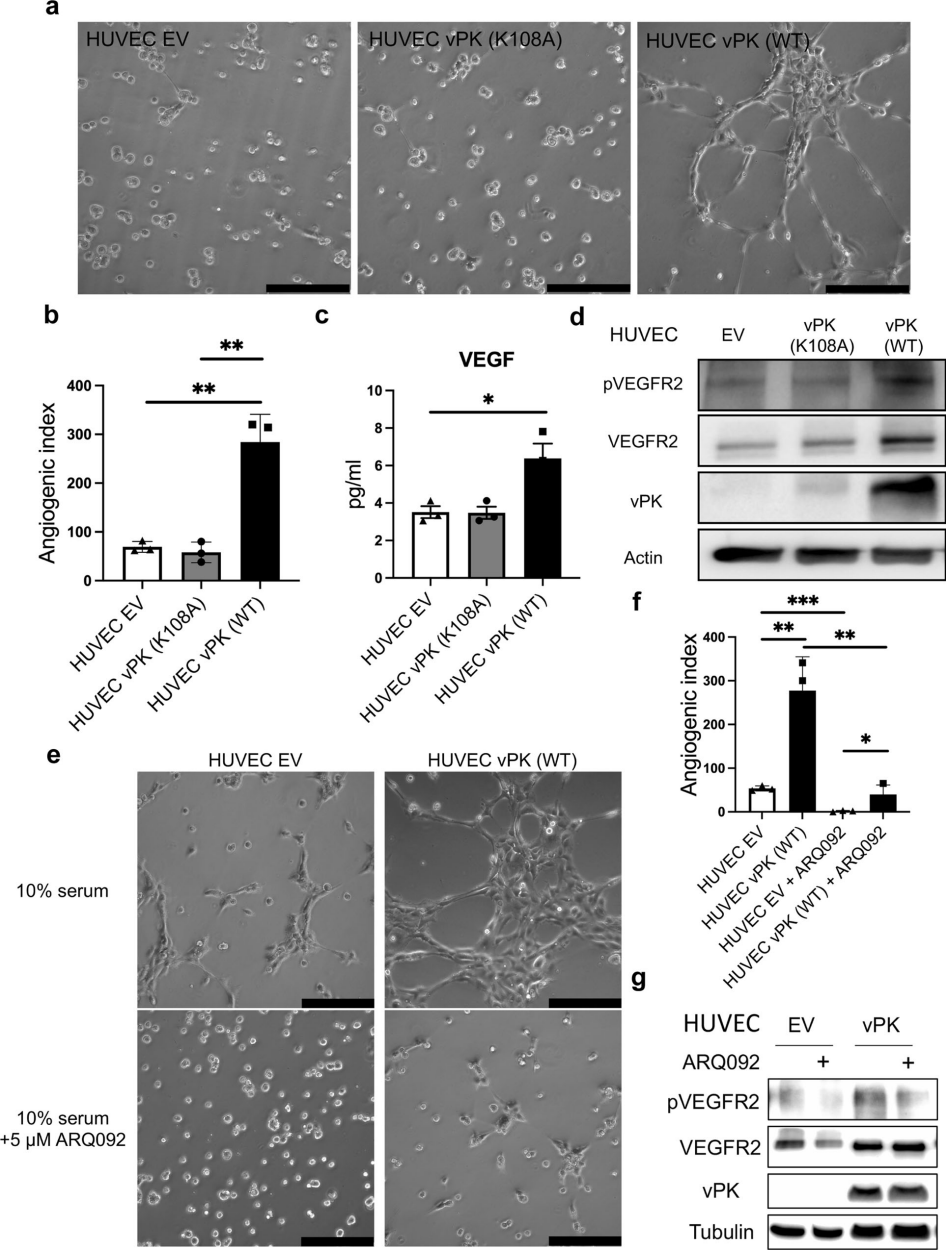
KSHV vPK (WT) augments tubule formation through the AKT signal pathway. **a** Angiogenesis assay using HUVECs expressing vPK 3h post-plating. Images are representative of four independent experiments (*n* = 3). Scare bars indicate 200 μm. **b** The relative angiogenesis index shown in panel **a** was quantitated by Image J software. **c** VEGF protein levels in the supernatants of HUVEC EV, HUVEC vPK (K108A), and HUVEC vPK (WT) cultured in 10% serum medium for 24 h. **d** Immunoblot of cell lysates from HUVEC EV, HUVEC vPK (K108A), and HUVEC vPK (WT) cultured in 10% serum medium for 24 h. **e** Representative images of tubule assay of cells pre-treated with or without 5 μM ARQ 092 for 24 h. The scale bar indicates 200 μm. **f** Image J quantified the angiogenic index of tubules shown in panel **e**. **g** AKT inhibitor (5 μM ARQ 092) inhibited phosphorylated VEGFR2 levels in both HUVEC EV and HUVEC vPK (WT) cells cultured in media containing 10% serum.

We used an endothelial tubule formation assay to model angiogenesis in vitro [25]. We observed a significant increase in tubule formation in HUVEC vPK (WT) cells, compared to the HUVEC EV at 3 h after plating (Fig. 6a, b). These results indicate that vPK promotes the angiogenic remodeling of HUVECs. However, the K108A mutation of vPK attenuates the angiogenic advantage of wild-type vPK (Fig. 6a, b). Vascular endothelial growth factor (VEGF) is important for endothelial cell growth and angiogenesis, and it is expressed in KS lesions [26]. Expression of vPK in HUVECs resulted in higher secreted VEGF levels (Fig. 6c) and the activation of the downstream VEGF receptor 2 (VEGFR2) signaling pathway (Fig. 6d), which was dependent on the catalytic activity of vPK.

Since AKT is downstream of VEGF/VEGFR2 [27] and we found increased activity of AKT in HUVEC vPK (WT) cells, we suspected that AKT1 also plays a vital role in upregulating angiogenesis in HUVEC vPK (WT) cells. We pre-treated HUVECs with ARQ 092 and found significantly decreased tubule formation and angiogenic index in HUVEC vPK (WT) cells though it was still higher than HUVEC EV cells (Fig. 6e, f). Treatment of cells with ARQ 092 decreased the levels of pVEGFR2 (Fig. 6g) and both phosphorylation sites of AKT1 (Supplementary Fig. S5). These findings suggest vPK can promote angiogenesis partly through the AKT pathway.

### vPK alleviates chemotherapy drug-induced toxicity in vitro and in vivo

**Fig. 7:**
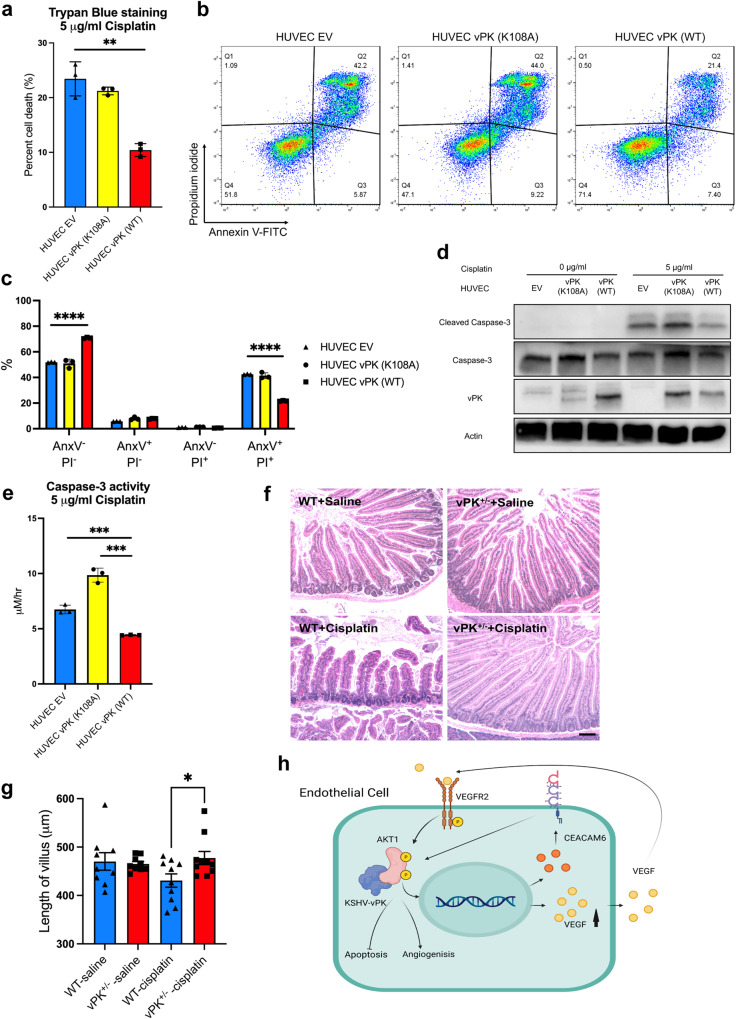
vPK expression prevents the cytotoxicity induced by cisplatin in vitro and in vivo. HUVECs were grown in complete medium and were treated with 5 μg/ml cisplatin for 24 h. **a** Cell viability was evaluated by trypan blue staining. **b** Cells were stained with Annexin V and PI and analyzed by FACS. **c** Quantitation of cells stained with Annexin V and PI. **d** Immunoblotting revealed cleaved Caspase-3 levels. Actin served as a loading control. **e** Caspase-3 activity induced by cisplatin was determined by colorimetric assay. All data are representative of three independent experiments (*n* = 3). **P < 0.01, and ***P < 0.001. **f** Representative hematoxylin and eosin-stained sections of the small intestine of WT and vPK^+/−^ mice after IP injection of saline or cisplatin (scale bar, 100 μm). **g** Quantification of the length of villi from mice with saline or cisplatin treatment. Three randomly selected fields from each mouse (*n* = 10) were quantified using LAS X. Two-tailed unpaired Student’s t-test was performed. *P < 0.05. **h** vPK activates the AKT pathway in multiple ways. vPK can bind to the PH domain of AKT1 and enhance AKT signaling by increasing the expression of CEACAM6 and VEGF/VEGFR2.

To further verify the effect of vPK on cell survival, we treated HUVEC cells expressing WT or mutnat vPK, as well as vPK transgenic mice [11] with the chemotherapy drug cisplatin. Cisplatin kills proliferating cells by interfering with DNA replication. HUVEC vPK (WT) cells had lower levels of cell death after treatment with cisplatin for 24 h, as measured by trypan blue staining, compared to HUVEC EV and HUVEC vPK (K108A) cells (Fig. 7a). Annexin V/PI staining also showed vPK (WT) expression decreases cell death by half (Fig. 7b, c). Cleaved Caspase-3 levels were lower in HUVEC vPK (WT) compared to EV and vPK (K108A) cells after treatment with cisplatin (Fig. 7d), which was concordant with Caspase-3 activity (Fig. 7e). Similar to previous results, the vPK K108A mutation abolished the survival advantage of HUVEC vPK (WT) subjected to cisplatin treatment. Cisplatin mouse models are widely used [28, 29] and cisplatin can damage the whole gastrointestinal tract in a dose-dependent manner [30]. To test whether vPK contributes to resistance to cisplatin, vPK transgenic mice were injected intraperitoneally with cisplatin (8 mg kg^−1^, day 1). Cisplatin severely disrupted the villi of the small intestine by day 5 in WT mice but not vPK^+/−^ mice as seen by the morphology and villi lengths (Fig. 7f, g). The immunohistochemistry stain for cleaved Caspase-3 in the small intestine of WT and vPK^+/−^ mice is shown in Supplementary Fig. S6. Thus, vPK also plays an important role in resistance of chemotherapy drug-induced toxicity in vivo.

## Discussion

KS is the most common malignancy in patients infected with HIV. Previous studies have suggested KSHV vPK mimics S6KB1 function and upregulates protein synthesis, proliferation, and vascularization [12]. In addition, aged transgenic vPK mice develop lymphoma at increased rates compared to matched WT mice [11]. Here we report that vPK inhibits apoptosis in response to different stimuli by upregulating AKT1 activity. We demonstrate that vPK binds to AKT1 through its PH domain and that the catalytic activity of vPK is necessary to confer an increased survival in cells.

Our findings provide insight into a novel function of vPK in regulating apoptosis triggered by extrinsic and intrinsic pathways. vPK also alleviates cisplatin-induced toxicity in vPK transgenic mice. AKT is known to promote cell survival. vPK’s activation of AKT1 likely contributes to this survival advantage phenotype. We found that both AKT inhibitors (ARQ 092 and MK2206) decreased the survival advantage of vPK (WT)-expressing cells. The canonical AKT1 pathway is an essential kinase hijacked by several viruses to prolong cell survival and prevent apoptosis [31]. KSHV vPK can constitutively activate the AKT1 pathway in endothelial cells through physical association with the PH domain (111-126 aa) of AKT1, which is important for autoinhibition [32]. Since vPK binds the PH domain of AKT1, we hypothesize that this can relieve autoinhibition of AKT1 resulting in its activation. The kinase-dead vPK also can bind AKT1 but it cannot activate the AKT signaling pathway, indicating that the kinase activity of vPK is essential for activating the AKT signaling pathway (Fig. 7h).

We also found that WT vPK’s induction of CEACAM6 and VEGF/VEGFR2 can activate the AKT signaling pathway (Fig. 7h) [22, 23, 27]. Expression of the kinase-dead vPK (K108A) protein [33] was not able to induce activation of AKT kinase compared to wild-type vPK or change the expression levels of CEACAM6 and VEGF/VEGFR2. These results indicate that the kinase activity of vPK is necessary to activate the AKT pathway. Other KSHV viral proteins have also been shown to activate the PI3K/AKT/mTOR signaling pathway [34].

We further report that vPK expression induces angiogenesis by activating VEGFR2-mediated AKT signaling in endothelial cells. We also found treatment with an AKT inhibitor significantly inhibits the angiogenic advantage in HUVEC vPK (WT) cells though they still had higher levels of angiogenesis compared to EV cells treated with an AKT inhibitor. This indicates vPK (WT) expression might activate other pathways important for angiogenesis. The c-Jun N-terminal kinase (JNK) and the mitogen-activated kinases (MKK4 and MKK7) are additional phosphorylation targets of vPK, and they also participate in angiogenesis [33, 35]. KSHV ORF36 vPK also activates the MAPK/ERK signaling pathway, which might also contribute to angiogenesis [33, 36].

Taken together, our results indicate that the KSHV viral kinase, vPK, activates the AKT1 signaling pathway, confers a cell survival advantage to endothelial cells, and induces angiogenesis.

## Materials and methods

### Cell lines and cell culture

Human telomerase reverse transcriptase (hTERT)-immortalized human umbilical vein endothelial cells (HUVECs) were cultured in Endothelial Cell Basal Medium 2 (PromoCell Inc., #C-22211) with their respective supplement kits (PromoCell Inc., #C-39211) with 10% FBS, 1% penicillin-streptomycin, and 2 mM L-glutamate. Human embryonic kidney-293T cells (HEK293T) were cultured in DMEM medium supplemented with 10% FBS, 1% penicillin-streptomycin, and 2 mM L-glutamate.

### Generation of HUVEC EV, vPK (K108A), and vPK (WT) stable cell lines

Retroviruses were generated using TK502-EV, TK502-3x-Flag-vPK (K108A), TK502-3x-Flag-vPK (WT) plasmids as described previously [12]. HEK293 cells stably expressing gag and pol were transfected with a plasmid containing VSV-G and a corresponding TK502 plasmid using Lipofectamine 2000 (Invitrogen, #11668019). Viral supernatants were collected three days after transfection. HUVECs were centrifuged with retrovirus at 2500 rpm for 90 min at 30 °C in serum-free media containing polybrene (10 µg/mL, Sigma, #107689). The next day, new complete media with 800 µg/mL of G418 (Life Technologies, #10131035) was added for selection.

### Cell growth curve

Approximately 50,000 HUVECs were seeded on six-well plates with Endothelial Cell Basal Medium 2 containing 10% serum (*n* = 3). Pictures (*n* = 9 fields per well) were taken every 24 h, and cell number was quantified over a time period of 72 h.

### Cell survival assay

HUVECs (3 × 10^5^) were plated in six-well plates and cultured in complete medium. After 24 h, HUVECs were washed with DPBS and cultured with medium without serum or supplements. Cell number was manually quantified from 10 different fields over a course of 5 days (*n* = 3).

### Annexin V-FITC/PI staining

Apoptosis was determined using the Annexin V-FITC/PI staining kit according to the manufacturer’s instructions (Biolegend, #640914). Samples were processed on a MACSQuant VYB flow cytometer (Miltenyi Biotec), and data were analyzed using FlowJo software. Apoptosis was evaluated by measuring the ratios of Annexin V^+^/PI^−^ cells and Annexin V^+^/PI^+^ cells (*n* = 3).

### SYTOX and Hoechst staining

Cells were counterstained with 1 µM Hoechst 33342 (Fisher Scientific, #H3570) and 0.5 µM SYTOX Green Nucleic Acid Stain (Thermo Scientific, #S7020) for 2 h (*n* = 3). Images were taken using a Leica DMi8 microscope with the same exposure time setting. The intensities of fluorescence and SYTOX/Hoechst positive ratios were analyzed with LAS X software.

### Compounds

The cells were treated as followed: Miransertib (or ARQ 092, 1 µM in 0% serum medium and 5 µM in 10% serum medium, Cayman Chemical Company, #21388); MK2206 (2 µM, Selleck Chemicals, #S1078). DMSO was used as a vehicle control in ARQ 092 and MK2206 experiments. 5 µg/ml of cisplatin was dissolved in medium by shaking for 1 h. Cells were treated with the compounds for 24 h before lysates were collected (*n* = 3).

### RNA isolation and qRT-PCR

Total RNA was isolated (RNeasy Plus Mini Kit, Qiagen, #74136), and cDNA was synthesized using 1 μg of RNA (iScript cDNA synthesis kit, Bio-Rad, #1725035). Then qRT-PCR was performed using a Quantstudio 6 Flex real-time PCR machine (Thermo Fisher) with 2 × SensiFAST SYBR Lo-Rox real-time PCR master mix (Thomas Scientific, #C755H96). CEACAM6, the forward primer 5′-GGCAACATGACCCTCACTCT-3′ and reverse primer 5′-GGAGCACTTCCAGAGACTGT-3′; Actin, the forward primer 5′-ACCTTCTACAATGAGCTGCG-3′ and reverse primer 5′-CCTGGATAGCAACGTACATGG-3′. Two-tailed Student’s T tests of ΔCt values were used to compare gene expression (*n* = 3).

### RNA-Seq

Total RNA was extracted from two sample groups-HUVEC EV and HUVEC vPK (WT) under three different serum concentrations (0%, 2%, and 10%) (*n* = 2). Genewiz (AZENTA Life Sciences, (https://www.genewiz.com/)) performed library preparation followed by RNA sequencing utilizing Illumina NovaSeq technology. GeneWiz performed the analysis for differential gene expression and volcano plot. Read counts within diverse sample libraries were normalized to account for factors like sequencing output. DESeq2 was used to identify genes with differential expression between HUVEC EV and HUVEC vPK (WT) samples. P-values and log2 fold changes were computed via the two-tailed Wald test. Differentially expressed genes were determined using thresholds of adjusted P-value < 0.05 and an absolute log2FoldChange > 1. The RNA-Seq data can be accessed on the NCBI Geo database using accession number GSE241970.

### Immunoblotting

Immunoblotting was performed as we previously described [25]. Membranes were blocked with 5% fat-free milk to prevent non-specific binding (or 5% BSA for cleaved Caspase-3) for 1 h and then incubated with the indicated antibodies overnight at 4 °C. The original Western blots are provided as supplementary material (*n* = 3).

### Antibodies

The following antibodies for Western blotting were used: Phospho-STAT3 (Y705) (Cell Signaling Technology [CST] #9145, 1:1000), Phospho-STAT3 (S727) (CST #9134, 1:1000), STAT3 (CST #9139, 1:1000), Phospho-AKT1 (T308) (CST #13038, 1:1000), Phospho-AKT1 (S473) (CST #3787, 1:1000), AKT (CST #9272, 1:1000), CEACAM6 (9A6) HRP (Santa Cruz Biotechnology, sc-59899, 1:500), Cleaved Caspase-3 (CST #9664, 1:1000), Caspase-3 (CST #9662, 1:1000), Caspase-7 (CST #9492, 1:1000), Cleaved Caspase-7 (CST #9491, 1:1000), anti-vPK antibody was used as described previously (1:1000) [37]. β-Actin conjugated with HRP (Santa Cruz Technology sc-47778, 1:3000), and secondary HRP-conjugated anti-rabbit (CST #7074, 1:2000) were also used.

### CellTiter-Glo (CTG) cell viability assay

Twenty thousand HUVECs were seeded into a 96-well plate for 24 h. Media was replaced with medium with or without 10% serum for 24 h. To quantify viability, 20 μl of CTG (Promega, #G7573) was added into each well and luminescence intensities were measured using a CLARIOstar plate reader (BMG Labtech).

### Trypan blue assay

HUVECs were either serum starved or treated with the indicated drugs for 24 h (*n* = 3). The percentage of dead cells was quantified using a hemacytometer.

### Plasmids

AKT plasmids were purchased from Addgene (pCDNA3-HA-AKT1 plasmid (Addgene, #73408), pcDNA3 Hygro HA AKT2 (Addgene, #16000), pcDNA3.1 AKT3 HA (CT-gfp) (Addgene, #27293)). AKT1 mutant plasmids (pCDNA3-HA-AKT1-aa1-149 (Addgene, #73410), pCDNA3-HA-AKT1-aa1-408 (Addgene, #73412), pCDNA3-HA-AKT1-aa120-433 (Addgene, #73411)) were gifts from Dr. Jie Chen [38]. V5-tagged vPK plasmid was generated using the Q5 Site-Directed Mutagenesis Kit (VWR, #E0554S) [12].

### Coimmunoprecipitation

Whole cells were lysed in ice-cold RIPA buffer containing protease inhibitor cocktails (Sigma-Aldrich, 11836145001) at 4 °C and centrifuged at max speed (15000 rpm) for 10 min. The lysates were incubated with the indicated beads (HA-antibody-conjugated beads (Thermo Scientific, #88836), Anti-V5-tag mAb-Magnetic Beads (VWR, #M167-11), or ANTI-FLAG® M2 Affinity Gel (Sigma-Aldrich, # A2220)) overnight at 4 °C. Beads were washed four times with RIPA buffer with protease inhibitor cocktails and boiled in an appropriate amount of 2× Laemmli sample buffer (Sigma-Aldrich, #S3401-10VL). Western blotting was performed as described above (*n* = 3). Membranes were probed with anti-V5 HRP (Thermo Fisher Scientific, #R961-25, 1:1000), anti-HA HRP (Cell Signaling Technology, #14031, 1:1000), or anti-Flag HRP (Sigma-Aldrich, #A8592, 1:3000) antibodies.

### Caspase-3 activity assay

Caspase-3 activity was measured using an ApoAlert Caspase-3 Fluorescent Assay Kit (Takara Bio, #630215, *n* = 3). Fluorescent intensities were measured using a CLARIOstar plate reader (BMG Labtech).

### Tubule formation assay

HUVECs (1.25 × 10^5^) in 1 ml of complete media were seeded on top of 300 μl of Matrigel (Corning, #354234) in a 24-well plate (*n* = 3). For the AKT1 inhibitor, ARQ 092, treatment, cells were pretreated with 5 μM of ARQ 092 for 24 h (*n* = 3). Five images were taken per well 3 h after seeding, and the angiogenic indexes (branching points) were measured using ImageJ.

### VEGF ELISA measurement

Supernatants from HUVECs were concentrated 10 times using Amicon Ultra-4 Centrifugal Filter Units (Ultracel, 3 KDa, EMD Millipore, #UFC800324), and measured by a Human VEGF Quantikine ELISA Kit (R&D systems, #DVE00, *n* = 3).

### vPK transgenic mice and cisplatin treatment

vPK transgenic C57BL/6 mice were described previously [11] and these mice develop a greater incidence of lymphoma. vPK^+/−^ mice were crossed with WT mice to generate matched controls of WT and vPK mice. Cisplatin was injected intraperitoneally into mice (8 mg kg^−1^, day 1) to test vPK’s function in chemotherapy drug resistance. Males and females were randomly chosen to be treated with saline or cisplatin. The small intestine was harvested on day 5 and fixed with 4% formalin. Histological assessment of small intestinal injury was carried out in a blinded fashion as previously described [28]. Three random fields of each mouse (*n* = 10, 5 males and 5 females, age around 8 weeks) were selected, and a total of 30 fields per group were evaluated by measuring the villi lengths (Leica DMi8 microscope and LAS X software).

### Immunohistochemistry

Small intestine tissue was harvested from male WT or vPK^+/−^ mice treated with cisplatin and placed in formalin (*n* = 5). Tissues were subsequently cut into thin slices and fixed in formalin containers for 72 h. Formalin-fixed tissues were then placed into Tissue-Tek® cartridges, rinsed with tap water, and stored in 70% ethanol. Formalin-fixed paraffin-embedded (FFPE) slides were then generated from each tissue sample with the aid of the Pathology Services Core Facility at the University of North Carolina at Chapel Hill.

For immunohistochemistry analyses, FFPE slides were baked at 65 °C for 1 h, and excess paraffin was removed. Tissue sections were rehydrated by subsequent incubations in histochoice (xylene substitute), 100% ethanol, and 95% ethanol. Antigens were unmasked by heating slides in a microwave submersed in 1× citrate buffer (CST) at high power until boiling, then heated on low power for an additional 10 min. Once cooled, slides were blocked with 3% hydrogen peroxide (Fisher) followed by 5% normal goat serum (CST). Tissue sections were incubated in a humidified chamber at 4 °C overnight with cleaved Caspase 3 antibody (CST #9579, 1:500) or isotype control (1:500, CST) diluted in SignalStain Antibody Diluent (CST). The following day, the antibody was removed and the tissues were incubated with SignalStain Boost IHC Detection Reagent (CST) for 30 min in a humidified chamber. SignalStain DAB (CST) was then used for antigen staining of both control (isotype) and experimental (cleaved Caspase 3) tissue sections. Tissues were counterstained with hematoxylin (CST) following the manufacturer’s instructions. Slides were dipped three times in hematoxylin to achieve the counterstain. Tissues were dehydrated by subsequent incubations in 95% ethanol, 100% ethanol, and histochoice. Tissue sections were then mounted with coverslips (Corning) using SignalStain Mounting Medium (CST) and slides were dried overnight. Images were acquired in one sitting using a Leica DMi8 inverted microscope with color camera and the LAS X software.

### Statistics

All experiments were performed in triplicate per treatment and repeated in three independent experiments; results are shown as mean ± SEM. All data was analyzed by two sided Student’s t-test (except cell survival over time which was analyzed using two-way ANOVA) using GraphPad Prism 9. P-values of <0.05 were considered statistically significant.

### Supplementary information

Supplementary Information

Original Western Blots

Checklist

## Data Availability

The RNA-Seq data can be accessed on the NCBI Geo database using accession number GSE241970. All original Western blots data have been uploaded as Supplementary material.
