# Supplementary material for: Kaposi’s sarcoma-associated herpesvirus viral protein kinase augments cell survival
Source: Cell Death Dis. 2023 Oct 18;14(10):688. doi: 10.1038/s41419-023-06193-1 (PMC10585003; doi:10.1038/s41419-023-06193-1)

Supplementary Figures

Supplementary Figure S1. vPK expression in HUVECs promotes pSTAT3 levels. (a) vPK expression in HUVECs did not increase cell proliferation over a 72h culture period. (b) The cell survival rate of HUVEC EV, HUVEC vPK (K108A), and HUVEC vPK (WT) treated with serum-free medium over time. *P < 0.05 vs HUVEC EV cells. (c) Western blot analysis of STAT3, Phospho-STAT3 (Y705), and Phospho-STAT3 (S727) in HUVECs in different serum concentrations (10, 2, and 0%). β-Actin was used as a loading control. pSTAT3 is known to be activated in KSHV-infected cells and was upregulated in HUVEC vPK (WT) compared to HUVEC EV and HUVEC vPK K108A cells. (d) Immunoblot of STAT3, Phospho-STAT3 (Y705), and Phospho-STAT3 (S727) in HUVECs under serum deprivation condition for 48h.

Supplementary Figure S2. **Ectopic expression of vPK (WT) in HUVEC increases phosphorylated levels of AKT1 under normal serum conditions and serum starved conditions.** Stable HUVEC single colonies were first plated in complete medium. The medium was changed to 10% serum or 0% serum for 24h. Immunoblots were performed for the indicated protein. Total Actin is shown as a loading control.

Supplementary Figure S3. BGLF4 interacts with AKT1. HA-AKT1 and FLAG-BGLF4 plasmids were transfected into HEK293T cells individually or together. The next day, cell lysates were immunoprecipitated with anti-HA (a) or anti-Flag (b) beads and then immunoblotted with the indicated antibodies.

Supplementary Figure S4. **Multiple sequence alignment of AKT1, AKT2, and AKT3 protein sequences.** Mismatched sequences in the PH domain are highlighted in the yellow box.

Supplementary Figure S5. Efficiency of AKT inhibitor (5 μM ARQ 092) in inhibiting phosphorylated AKT1 levels in HUVECs cultured in media containing 10% serum.

Supplementary Figure S6. Immunohistochemistry analysis of cleaved Caspase 3 expression in the small intestines of cisplatin-treated WT and vPK$^{+/-}$ mice. Tissue sections taken from the small intestines of male WT and vPK$^{+/-}$ mice treated with cisplatin were subjected to immunohistochemistry for cleaved Caspase 3. Each sample slide contained two sequential tissue sections, one which was incubated with cleaved Caspase 3 antibody and one which was incubated with isotype control antibody for background staining. Tissue sections were developed with DAB and counterstained with hematoxylin. Images were acquired using a Leica DMi8 inverted microscope and color camera. Scale bar = 100 μm.

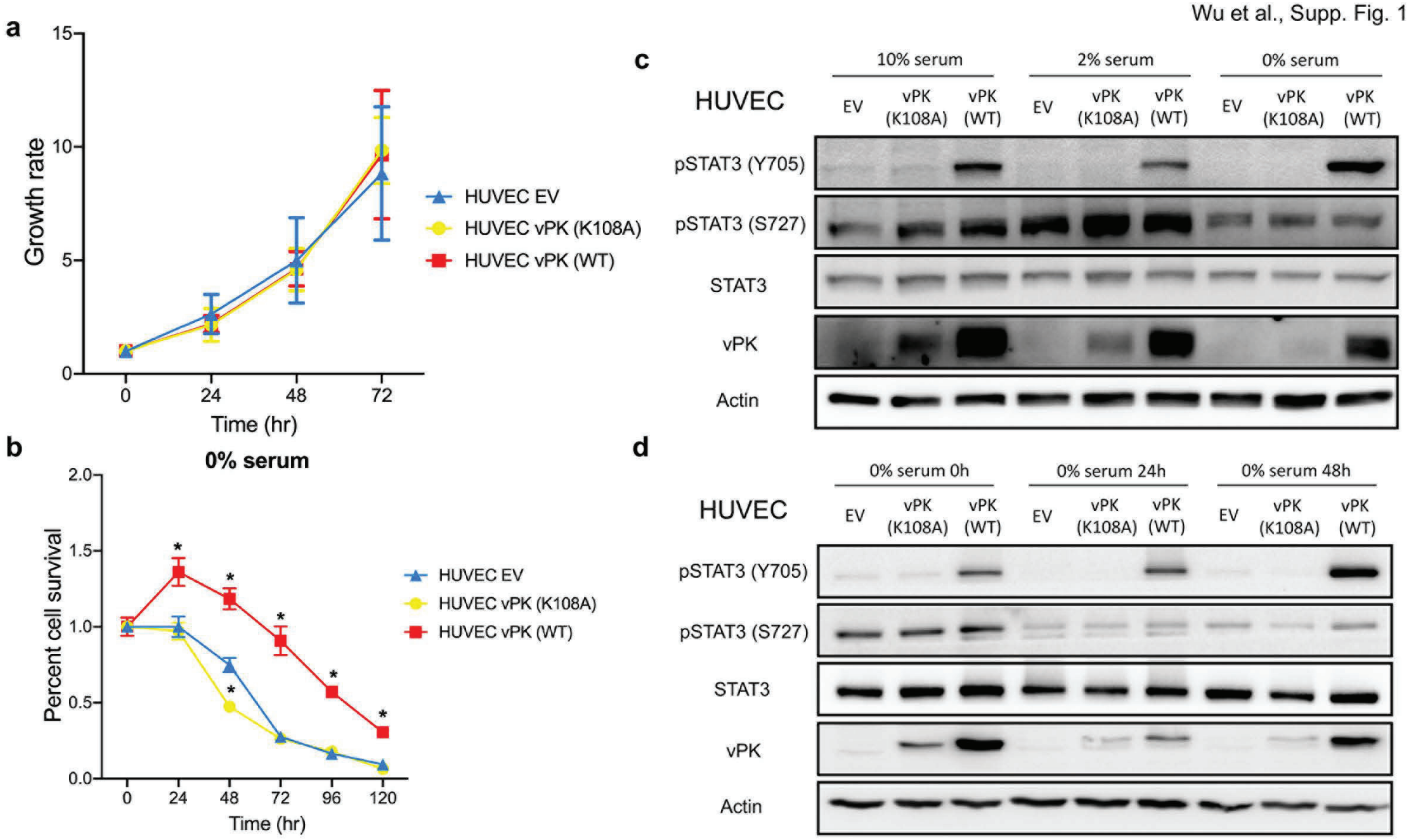

a
Growth rate
Time (hr)
HUVEC EV
HUVEC vPK (K108A)
HUVEC vPK (WT)
b
0% serum
Percent cell survival
Time (hr)
HUVEC EV
HUVEC vPK (K108A)
HUVEC vPK (WT)
c
HUVEC
10% serum
2% serum
0% serum
EV
vPK (K108A)
vPK (WT)
pSTAT3 (Y705)
pSTAT3 (S727)
STAT3
vPK
Actin
d
HUVEC
0% serum 0h
0% serum 24h
0% serum 48h
EV
vPK (K108A)
vPK (WT)
pSTAT3 (Y705)
pSTAT3 (S727)
STAT3
vPK
Actin

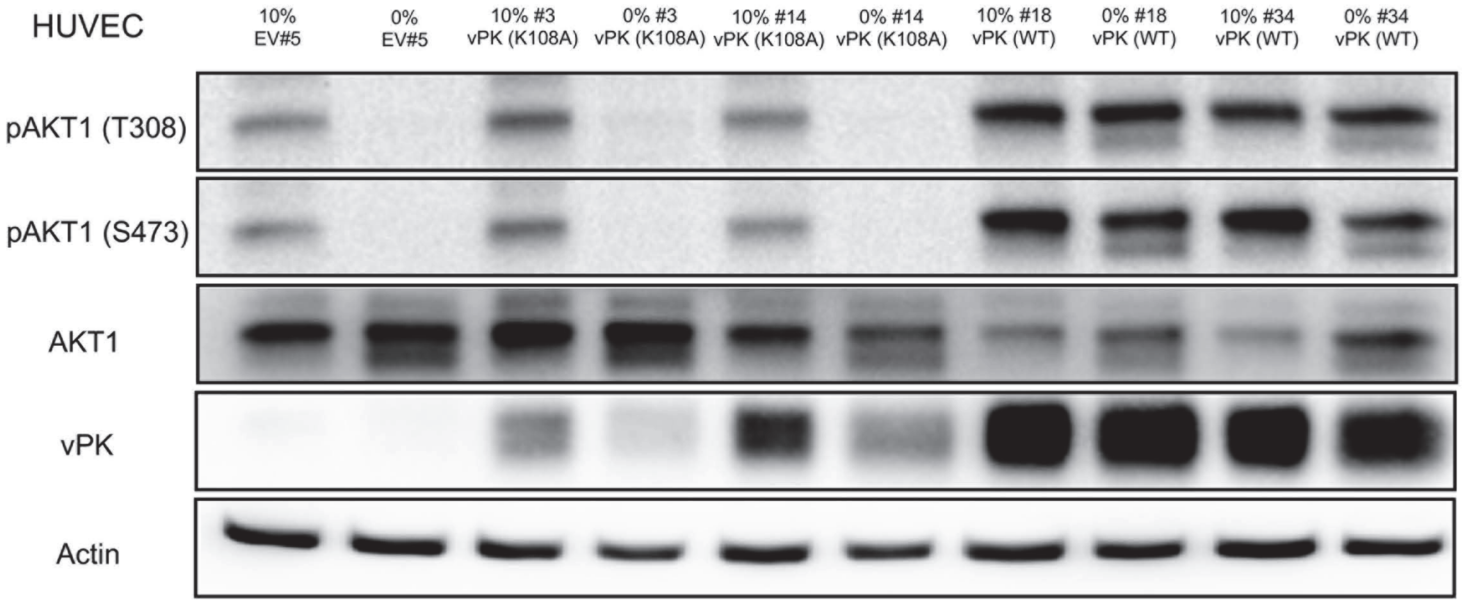
HUVEC
10% EV#5
0% EV#5
10% #3 vPK (K108A)
0% #3 vPK (K108A)
10% #14 vPK (K108A)
0% #14 vPK (K108A)
10% #18 vPK (WT)
0% #18 vPK (WT)
10% #34 vPK (WT)
0% #34 vPK (WT)
pAKT1 (T308)
pAKT1 (S473)
AKT1
vPK
Actin

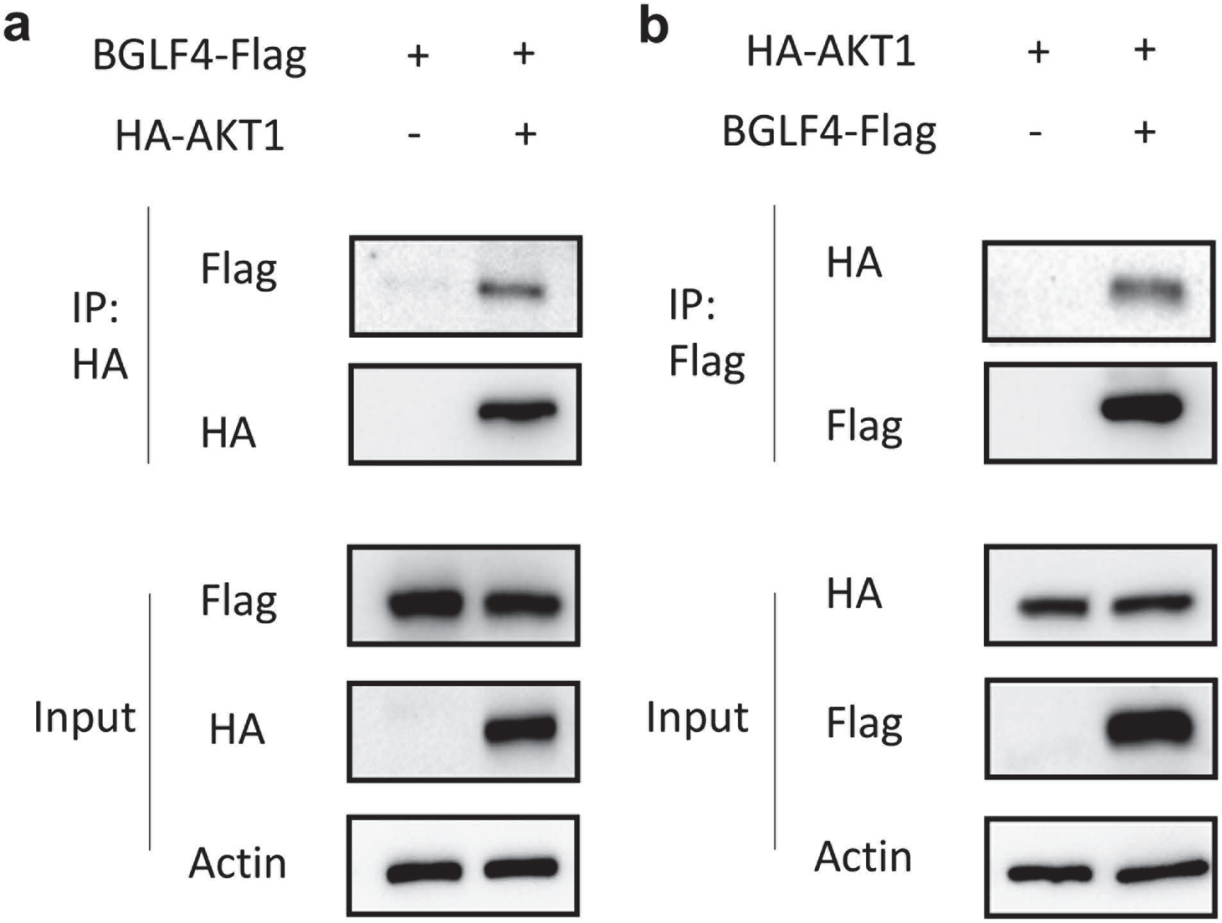
a
BGLF4-Flag + +
HA-AKT1 - +
IP: HA
Flag
HA
Input
Flag
HA
Actin
b
HA-AKT1 + +
BGLF4-Flag - +
IP: Flag
HA
Flag
Input
HA
Flag
Actin

N=3

Wu et al., Supp. Fig. 4

```
CLUSTAL O(1.2.4) multiple sequence alignment


sp|P31749|AKT1_HUMAN      MSDVAIVKEGWLHKRGEYIKTWRPRYFLLKNDGTFIGYKERPQDVDQREAPLNNFSVAQC   60
sp|P31751|AKT2_HUMAN      MNEVSVIKEGWLHKRGEYIKTWRPRYFLLKSDGSFIGYKERPEAPDQTLPPLNNFSVAEC   60
sp|Q9Y243|AKT3_HUMAN      MSDVTIVKEGWVQKRGEYIKNWRPRYFLLKTDGSFIGYKEKPQDVDL-PYPLNNFSVAKC   59
                          *.:*:::****::*******.*********.**:******:*:  *    ********:*

sp|P31749|AKT1_HUMAN      QLMKTERPRPNTFIIRCLQWTTVIERTFHVETPEEREEWTTAIQTVADGLKKQ--EEEEM   118
sp|P31751|AKT2_HUMAN      QLMKTERPRPNTFVIRCLQWTTVIERTFHVDSPDEREEWMRAIQMVANSLKQRAPGEDPM   120
sp|Q9Y243|AKT3_HUMAN      QLMKTERPKPNTFIIRCLQWTTVIERTFHVDTPEEREEWTEAIQAVADRLQRQ--EEERM   117
                          ********:****:****************::*:*****  *** **: *:::   *: *

sp|P31749|AKT1_HUMAN      DFRSGSPSDNSGAEEMEVSLAKPKHRVTMNEFEYLKLLGKGTFGKVILVKEKATGRYYAM   178
sp|P31751|AKT2_HUMAN      DYKCGSPSDSSTTEEMEVAVSKARAKVTMNDFDYLKLLGKGTFGKVILVREKATGRYYAM   180
sp|Q9Y243|AKT3_HUMAN      NCSPTSQIDNIGEEEMDASTTH-HKRKTMNDFDYLKLLGKGTFGKVILVREKASGKYYAM   176
                          :    *  *.   ***:.: :: : :  ***:*:****************:***:*:****

sp|P31749|AKT1_HUMAN      KILKKEVIVAKDEVAHTLTENRVLQNSRHPFLTALKYSFQTHDRLCFVMEYANGGELFFH   238
sp|P31751|AKT2_HUMAN      KILRKEVIIAKDEVAHTVTESRVLQNTRHPFLTALKYAFQTHDRLCFVMEYANGGELFFH   240
sp|Q9Y243|AKT3_HUMAN      KILKKEVIIAKDEVAHTLTESRVLKNTRHPFLTSLKYSFQTKDRLCFVMEYVNGGELFFH   236
                          ***:****:********:**.***:*:******:***:***:*********.********

sp|P31749|AKT1_HUMAN      LSRERVFSEDRARFYGAEIVSALDYLHSEKNVVYRDLKLENLMLDKDGHIKITDFGLCKE   298
sp|P31751|AKT2_HUMAN      LSRERVFTEERARFYGAEIVSALEYLHSRD-VVYRDIKLENLMLDKDGHIKITDFGLCKE   299
sp|Q9Y243|AKT3_HUMAN      LSRERVFSEDRTRFYGAEIVSALDYLHSGK-IVYRDLKLENLMLDKDGHIKITDFGLCKE   295
                          *******:*:*:***********:**** . :****:***********************

sp|P31749|AKT1_HUMAN      GIKDGATMKTFCGTPEYLAPEVLEDNDYGRAVDWWGLGVVMYEMMCGRLPFYNQDHEKLF   358
sp|P31751|AKT2_HUMAN      GISDGATMKTFCGTPEYLAPEVLEDNDYGRAVDWWGLGVVMYEMMCGRLPFYNQDHERLF   359
sp|Q9Y243|AKT3_HUMAN      GITDAATMKTFCGTPEYLAPEVLEDNDYGRAVDWWGLGVVMYEMMCGRLPFYNQDHEKLF   355
                          **.*.****************************************************:**

sp|P31749|AKT1_HUMAN      ELILMEEIRFPRTLGPEAKSLLSGLLKKDPKQRLGGGSEDAKEIMQHRFFAGIVWQHVYE   418
sp|P31751|AKT2_HUMAN      ELILMEEIRFPRTLSPEAKSLLAGLLKKDPKQRLGGGPSDAKEVMEHRFFLSINWQDVVQ   419
sp|Q9Y243|AKT3_HUMAN      ELILMEDIKFPRTLSSDAKSLLSGLLIKDPNKRLGGGPDDAKEIMRHSFFSGVNWQDVYD   415
                          ******:*:*****. :*****:*** ***::***** .****:*.* ** .: **.* :

sp|P31749|AKT1_HUMAN      KKLSPPFKPQVTSETDTRYFDEEFTAQMITITPPDQ--DDSMECVDSERRPHFPQFSYSA   476
sp|P31751|AKT2_HUMAN      KKLLPPFKPQVTSEVDTRYFDDEFTAQSITITPPDRYDSLGL--LELDQRTHFPQFSYSA   477
sp|Q9Y243|AKT3_HUMAN      KKLVPPFKPQVTSETDTRYFDEEFTAQTITITPPEKYDEDGMDCMDNERRPHFPQFSYSA   475
                          *** **********.******:***** ******::  . .: :: ::* *********

sp|P31749|AKT1_HUMAN      SGTA   480
sp|P31751|AKT2_HUMAN      SIRE   481
sp|Q9Y243|AKT3_HUMAN      SGRE   479
                          *
```

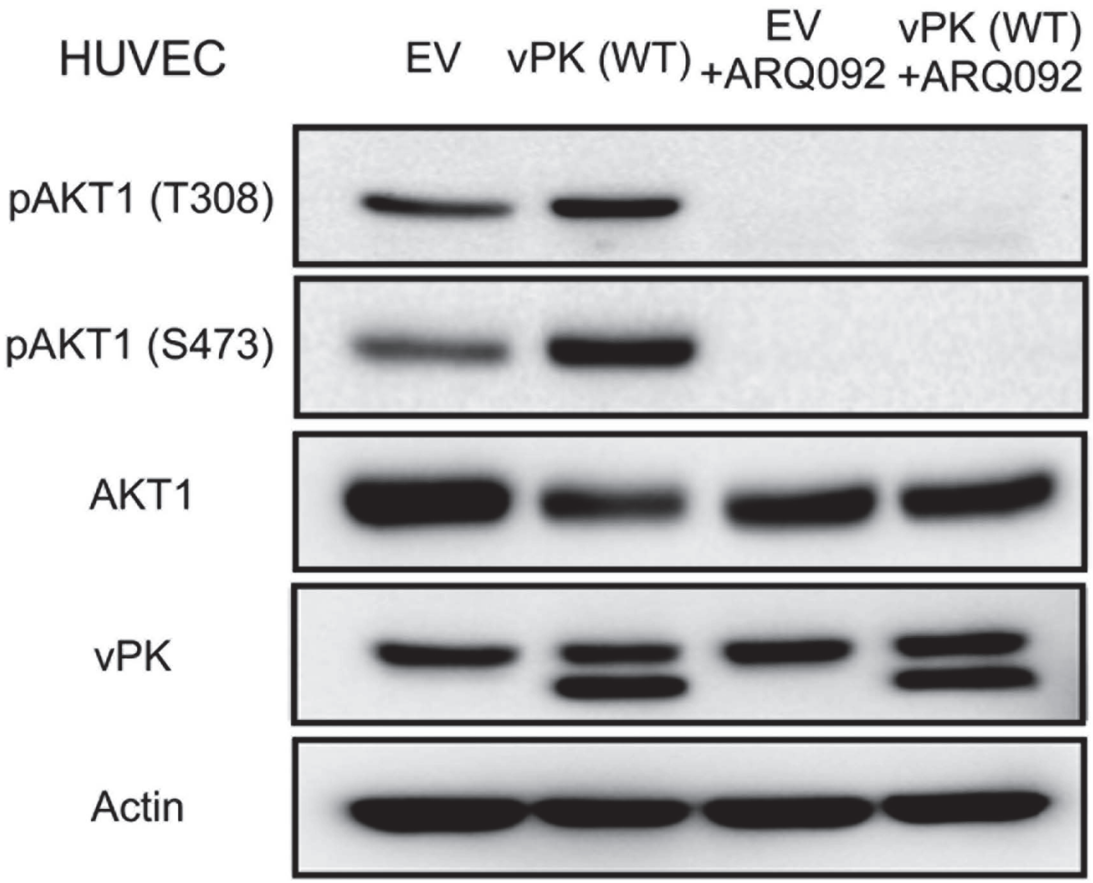
HUVEC
EV
vPK (WT)
EV
+ARQ092
vPK (WT)
+ARQ092
pAKT1 (T308)
pAKT1 (S473)
AKT1
vPK
Actin

Wu et al., Supp. Fig. 6

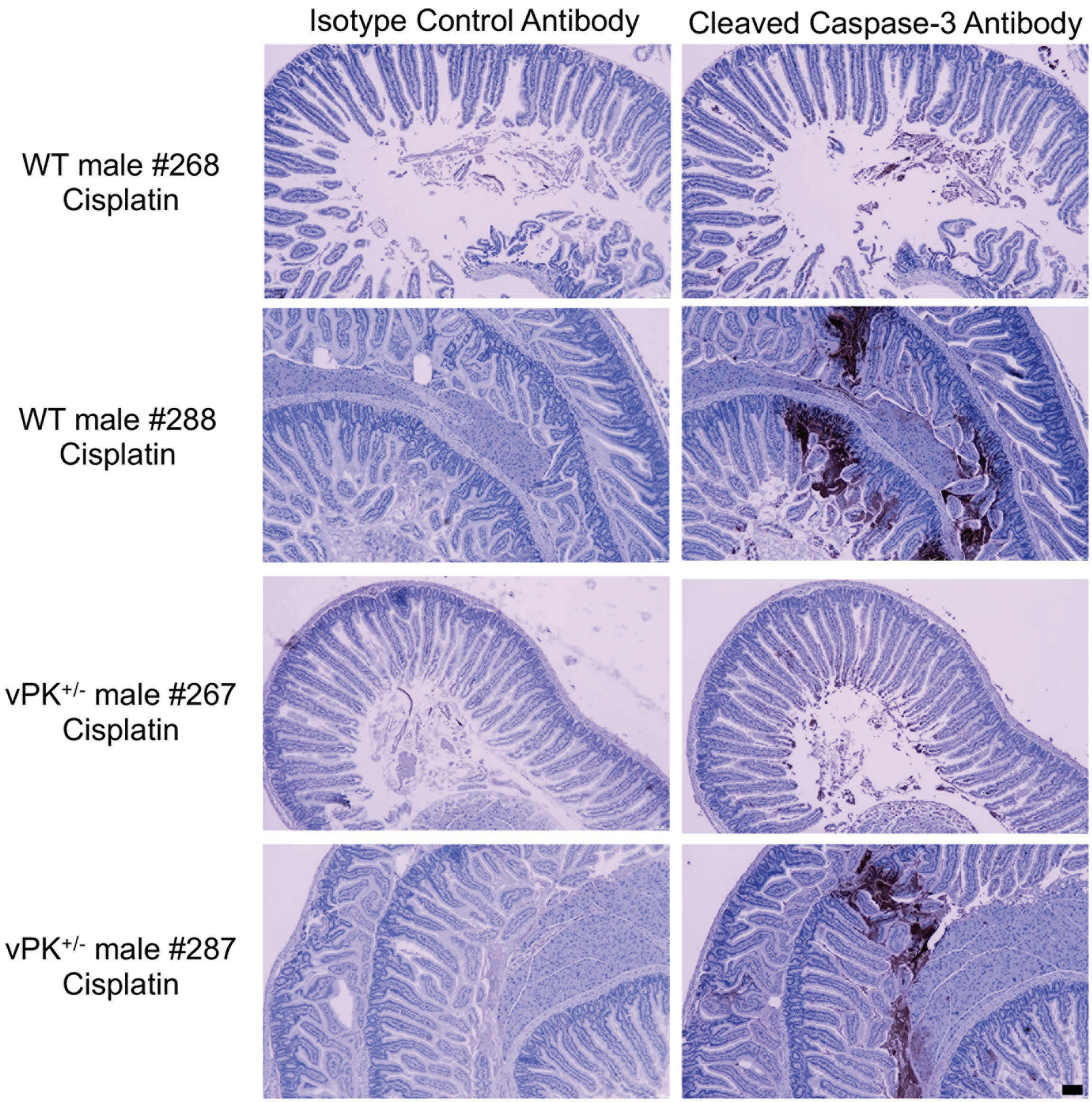

Supplement: Supplementary file 1 — Supplementary Information [file 41419_2023_6193_MOESM1_ESM.pdf]
