# Supplementary material for: Kaposi’s sarcoma-associated herpesvirus viral protein kinase augments cell survival
Source: Cell Death Dis. 2023 Oct 18;14(10):688. doi: 10.1038/s41419-023-06193-1 (PMC10585003; doi:10.1038/s41419-023-06193-1)

Figure 1i

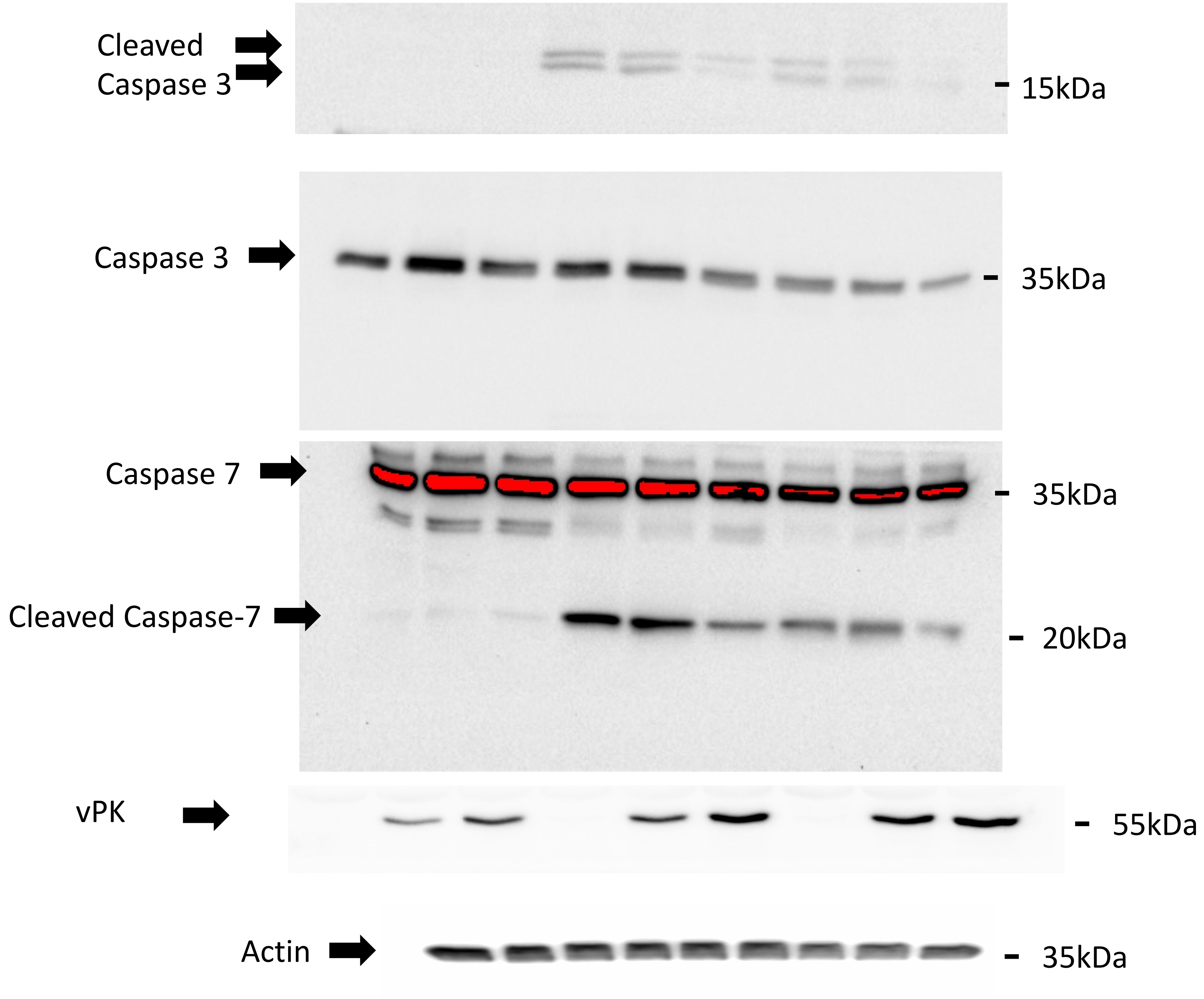
Cleaved Caspase 3
15kDa
Caspase 3
35kDa
Caspase 7
35kDa
Cleaved Caspase-7
20kDa
vPK
55kDa
Actin
35kDa

Figure 2f

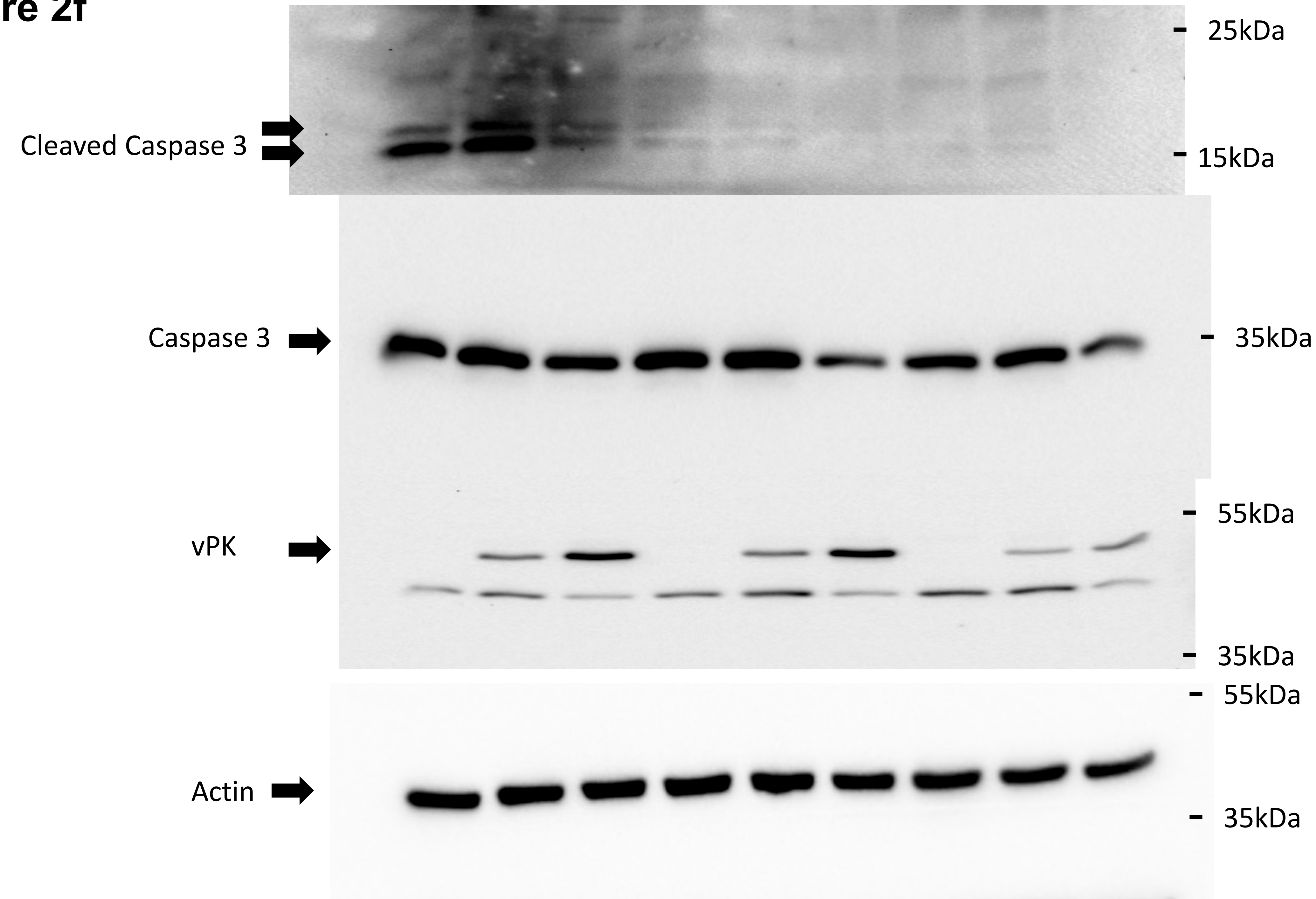
Cleaved Caspase 3
25kDa
15kDa
Caspase 3
35kDa
vPK
55kDa
35kDa
Actin
55kDa
35kDa

Figure 2I

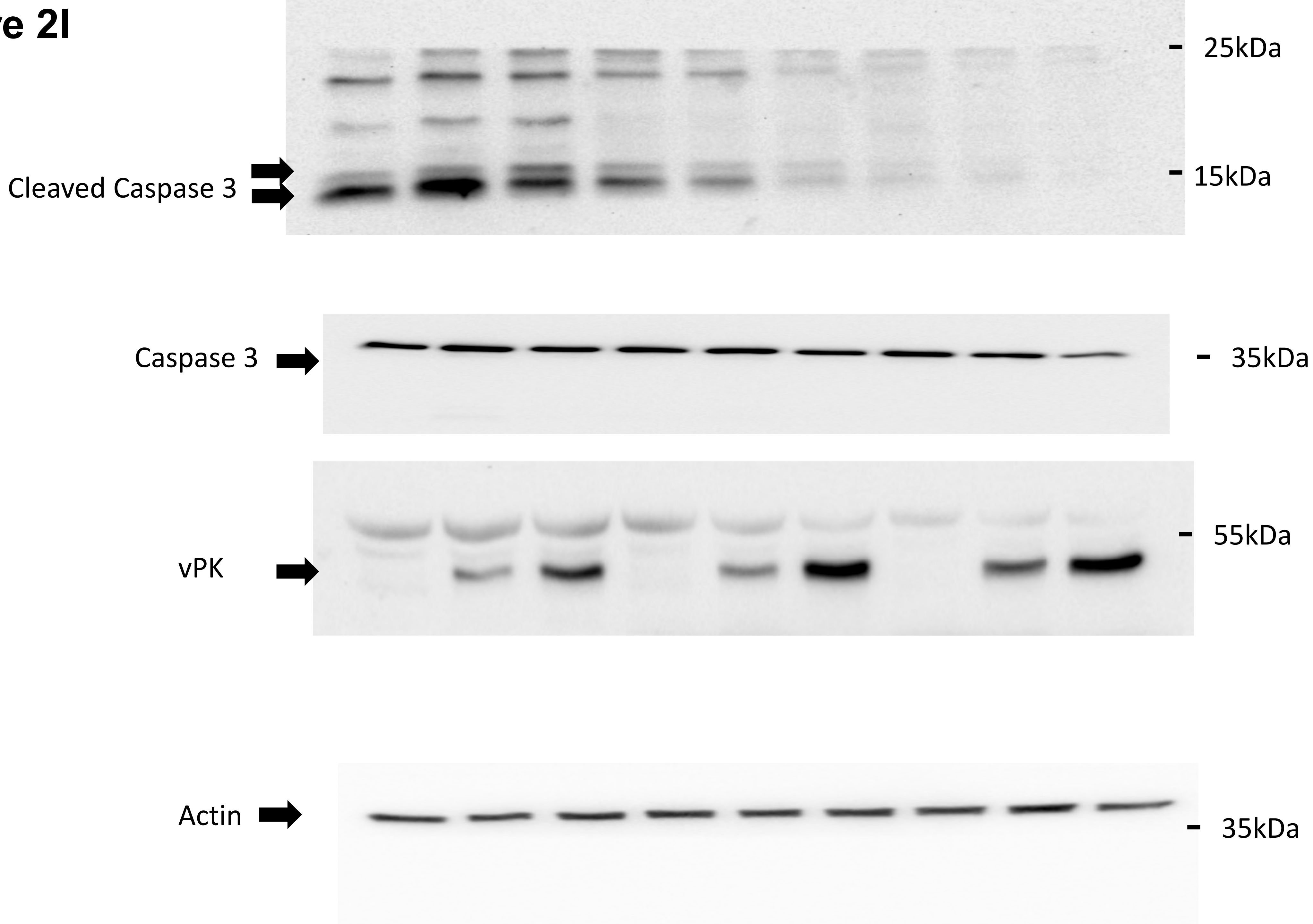

Cleaved Caspase 3
25kDa
15kDa
Caspase 3
35kDa
vPK
55kDa
Actin
35kDa

Figure 3e

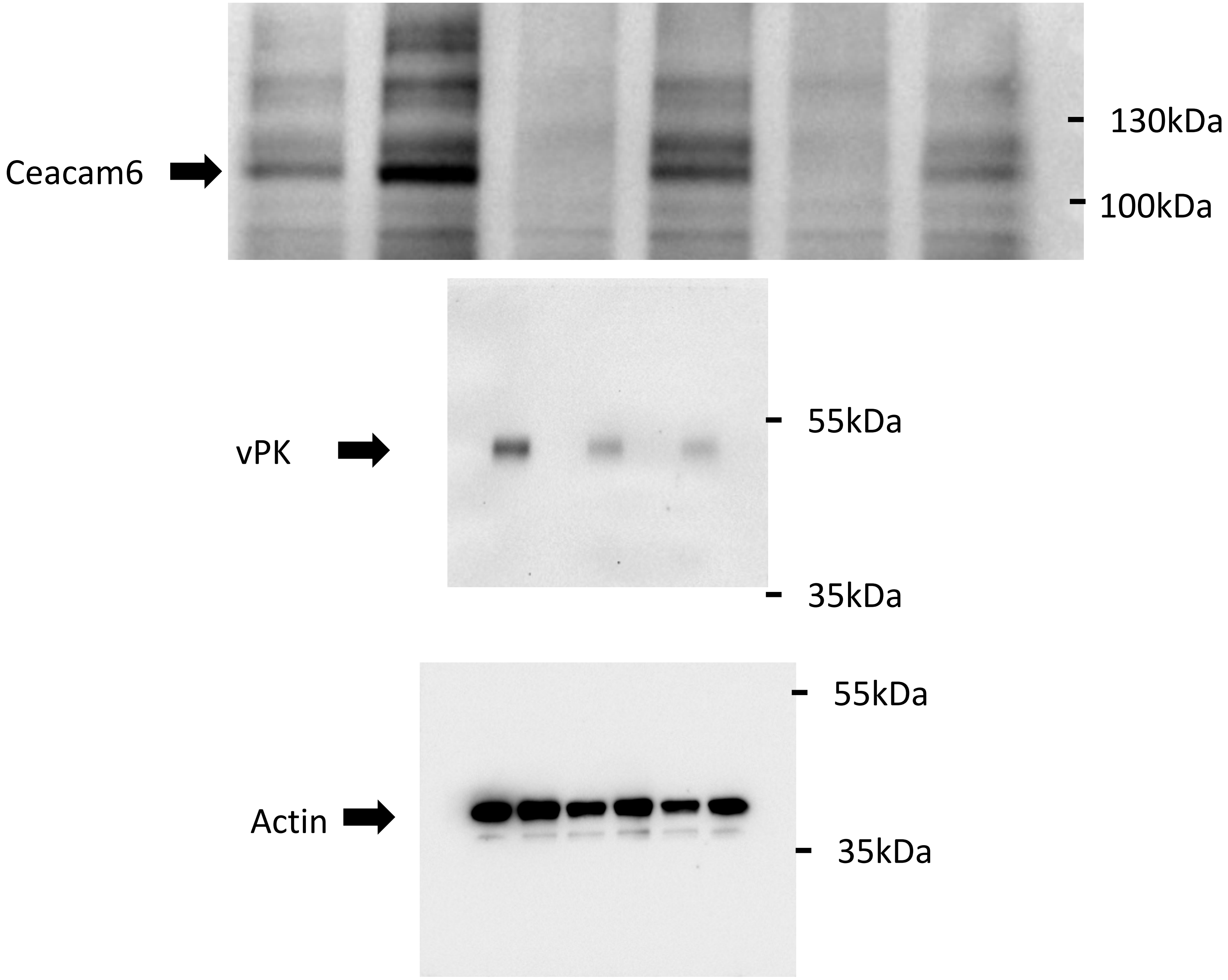

Figure 3f

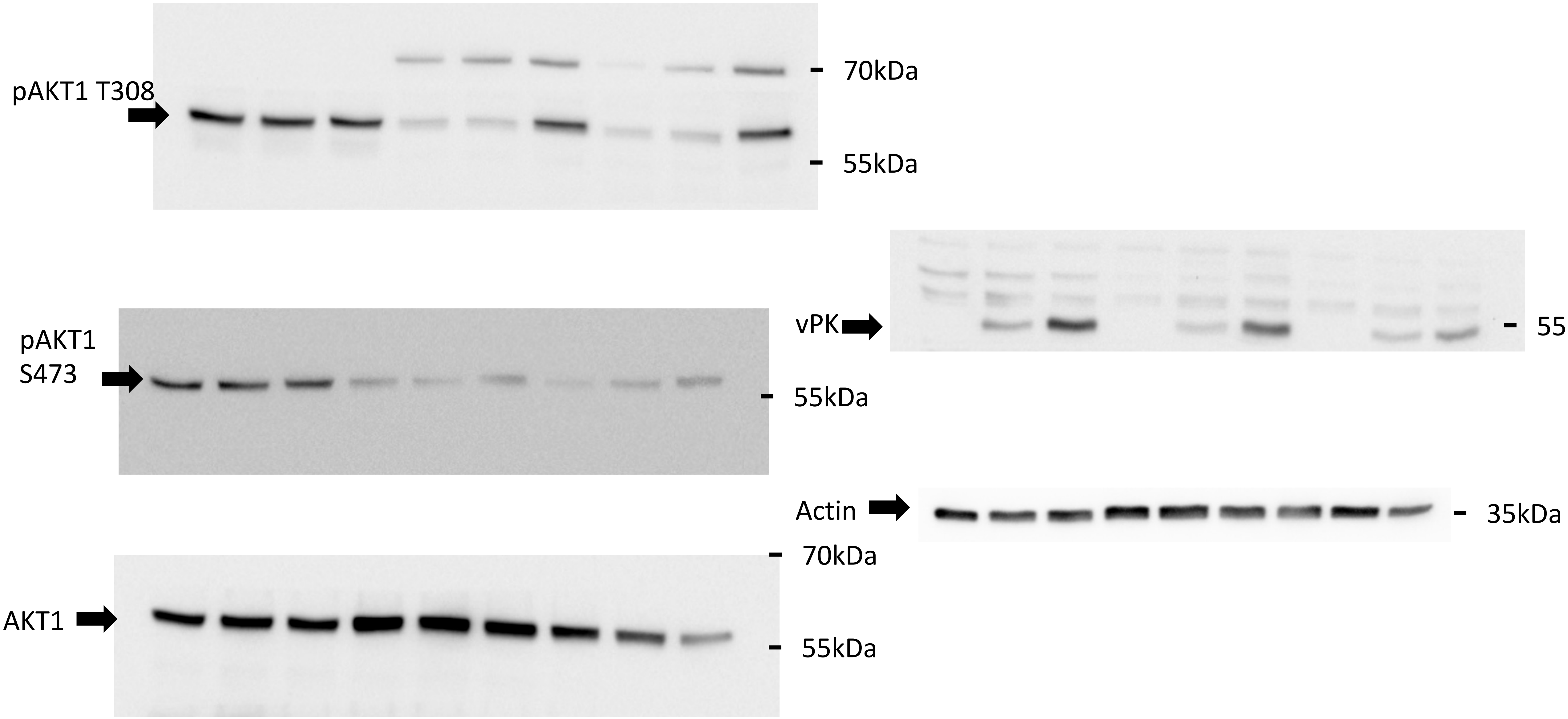

pAKT1 T308
70kDa
55kDa
pAKT1
S473
55kDa
AKT1
70kDa
55kDa
vPK
55
Actin
35kDa

Figure 3g

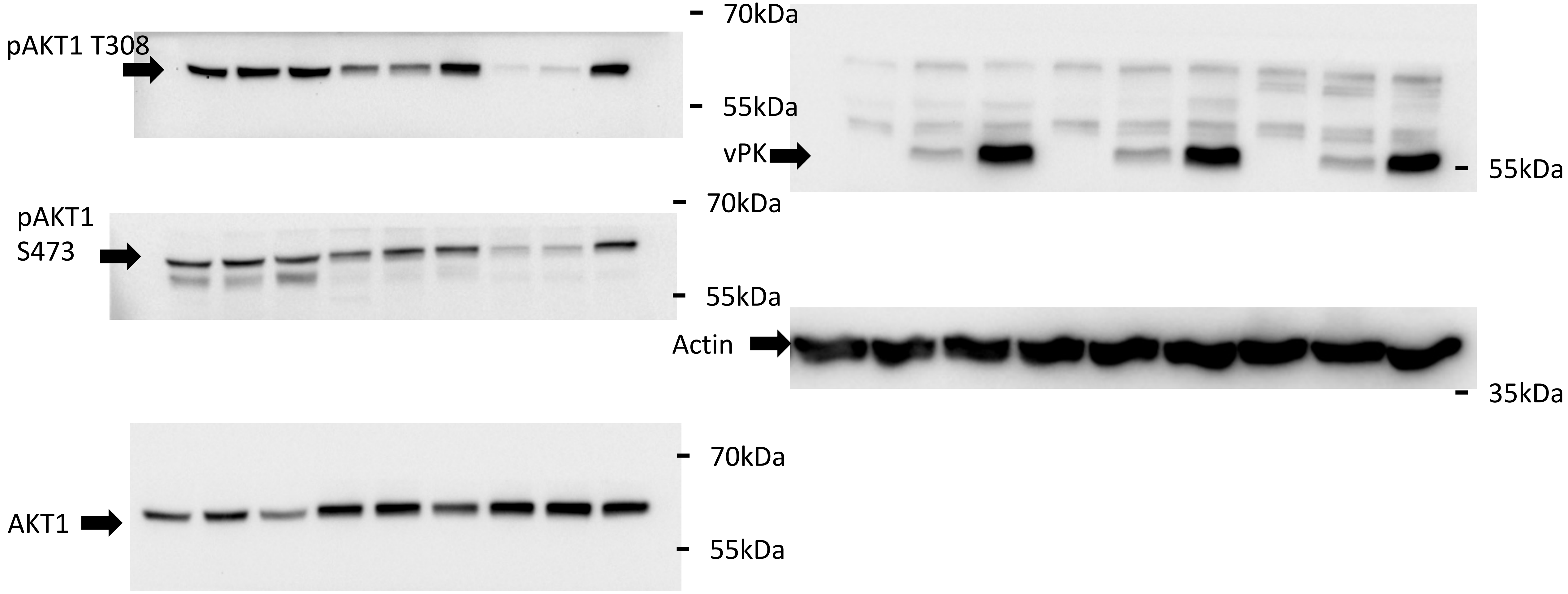

pAKT1 T308
70kDa
55kDa
vPK
55kDa
pAKT1
S473
70kDa
55kDa
Actin
35kDa
AKT1
70kDa
55kDa

Figure 3h

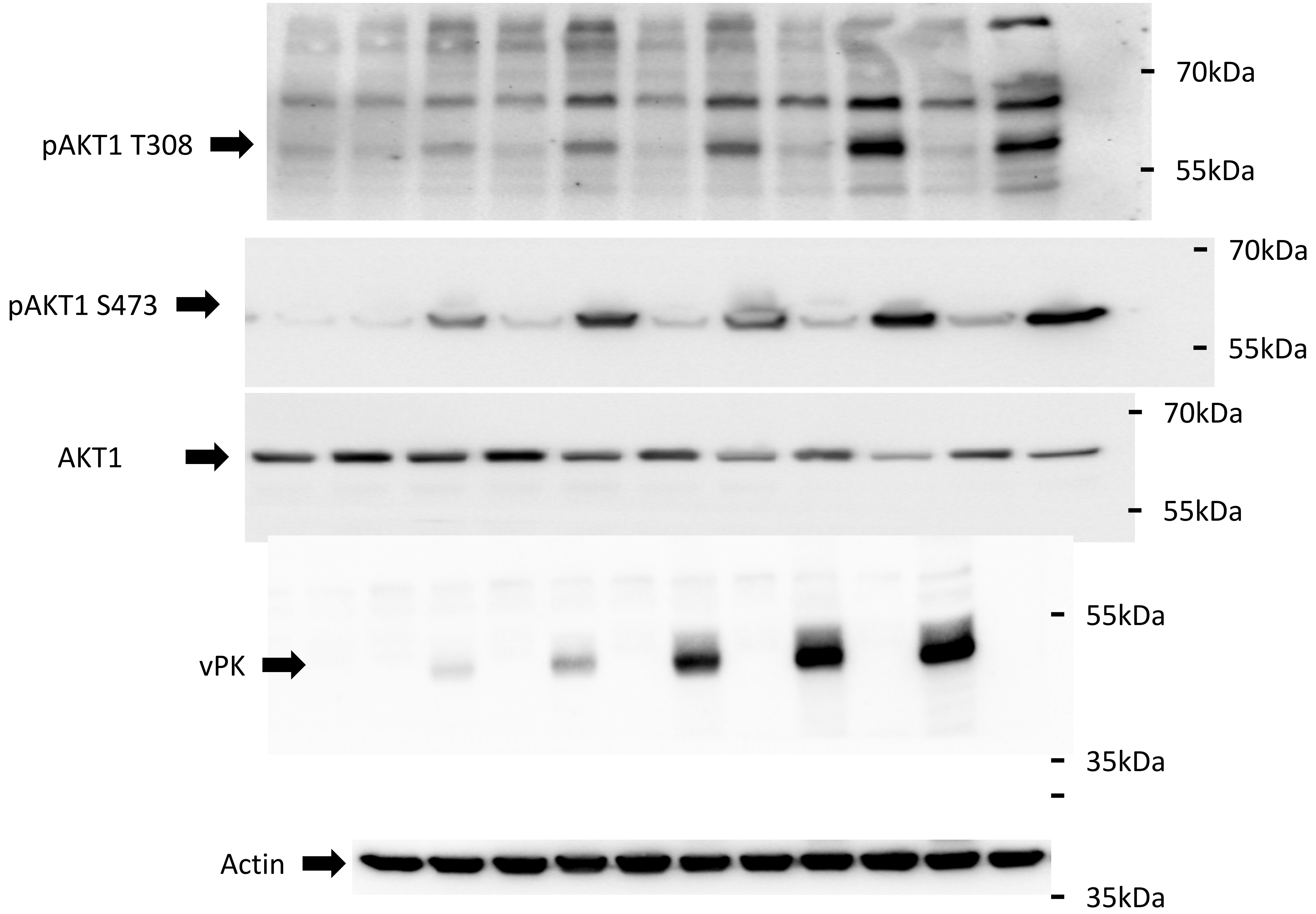

pAKT1 T308
70kDa
55kDa
pAKT1 S473
70kDa
55kDa
AKT1
70kDa
55kDa
vPK
55kDa
35kDa
Actin
35kDa

Figure 4a and 4b

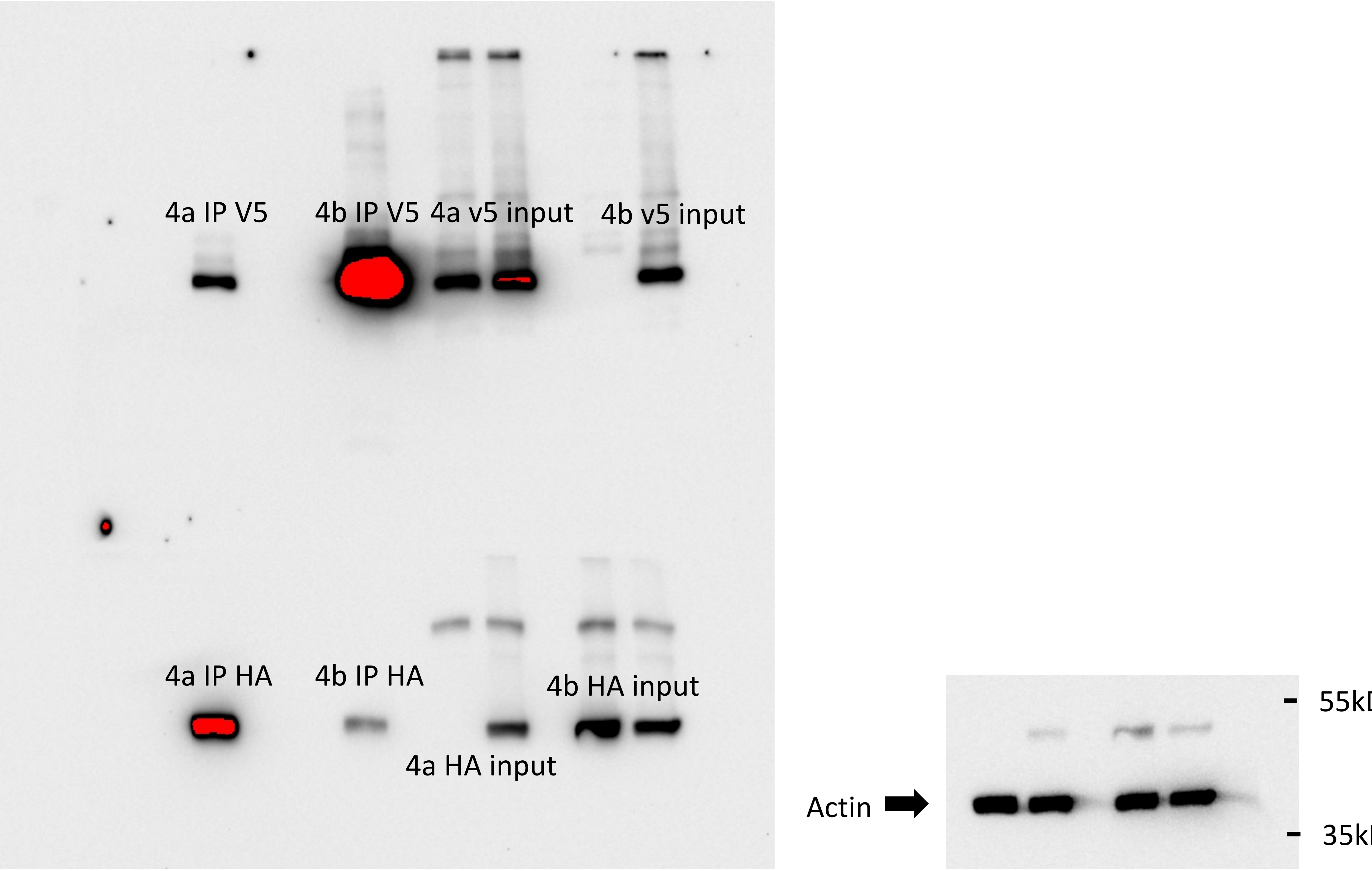

Figure 4e

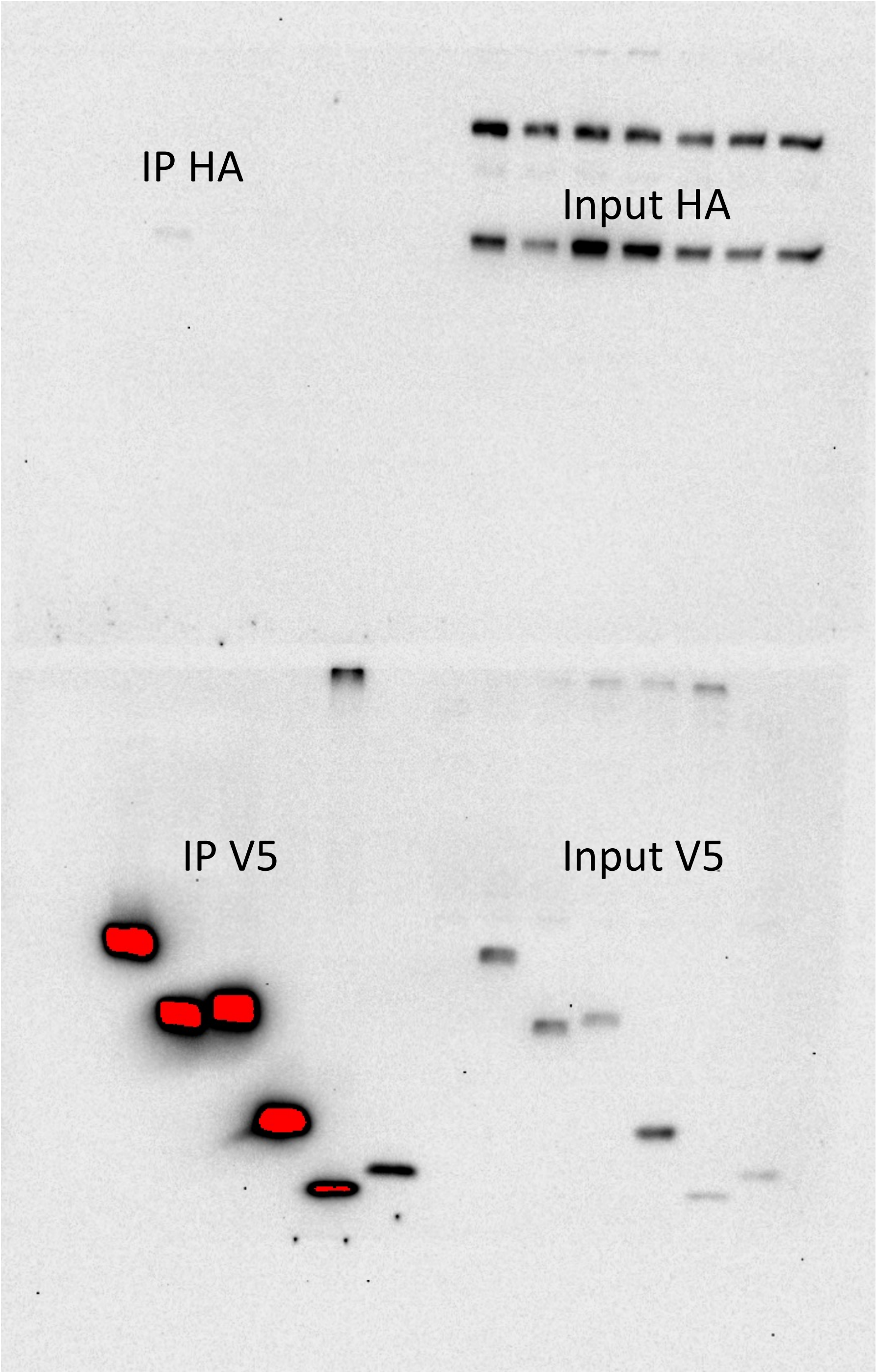

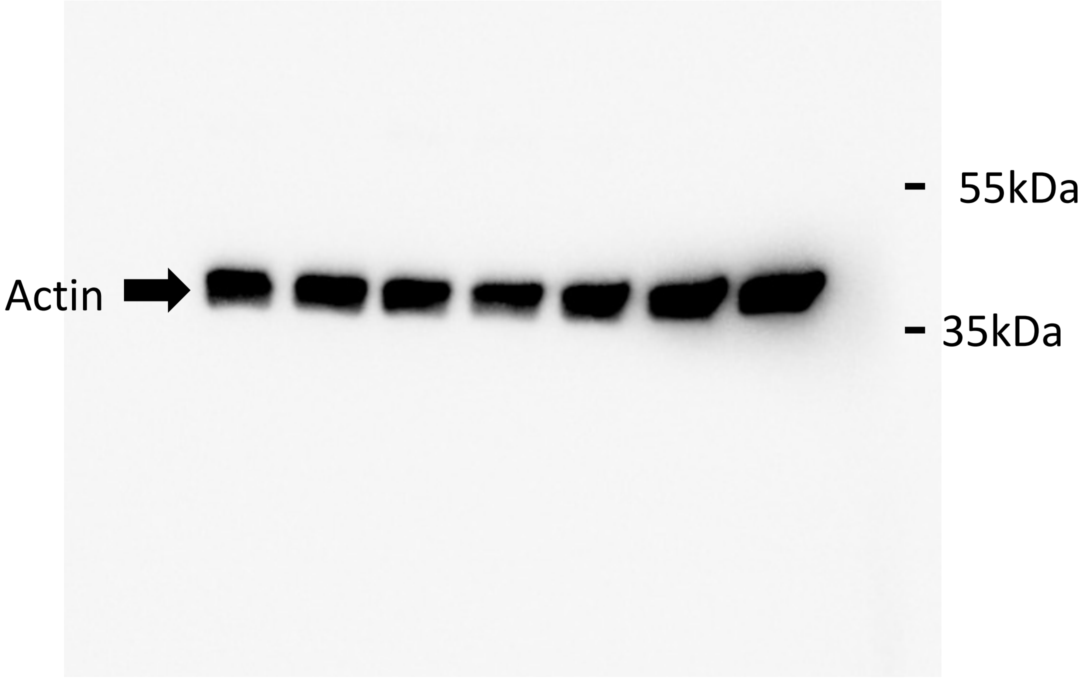

Figure 4f and 4g

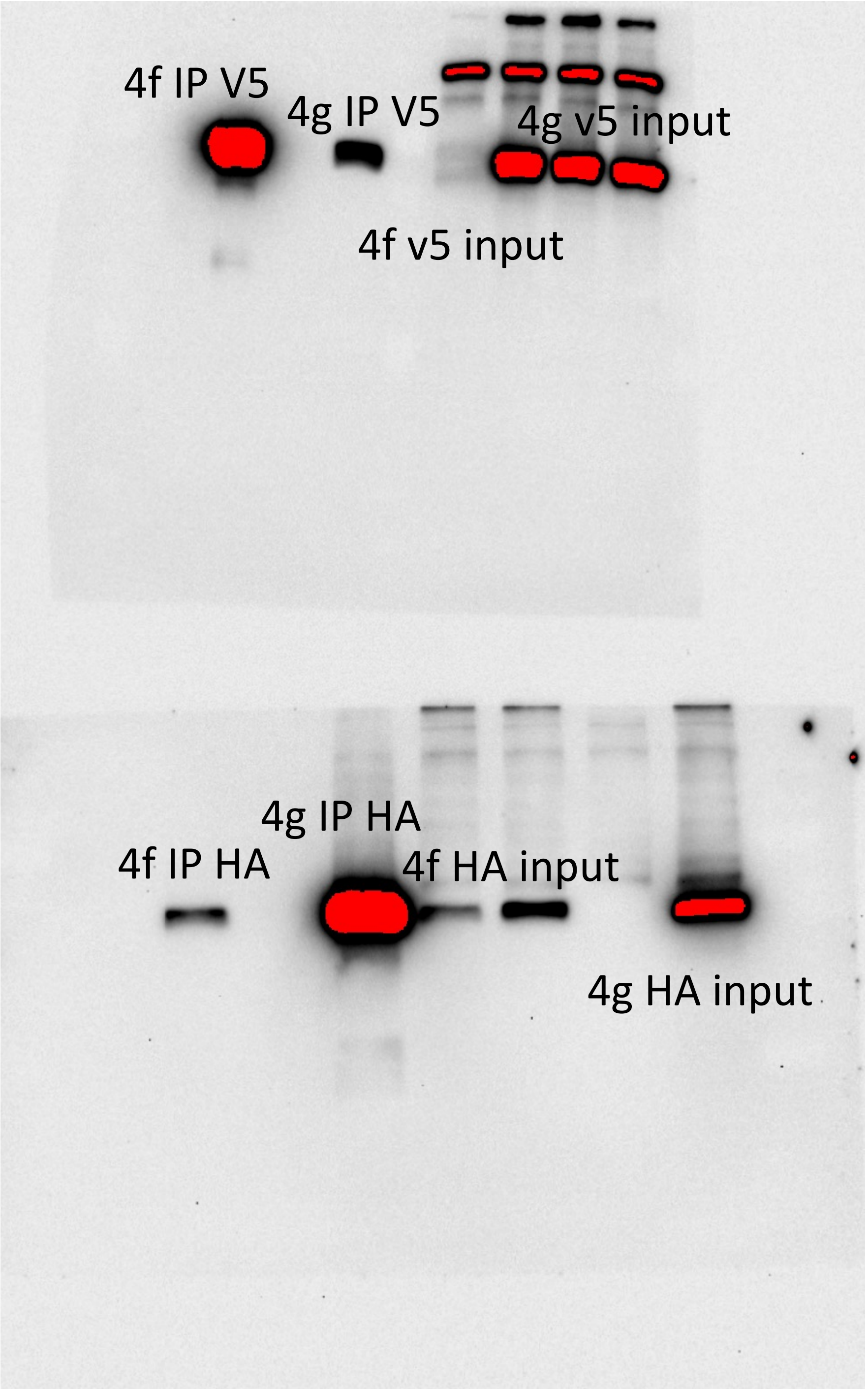

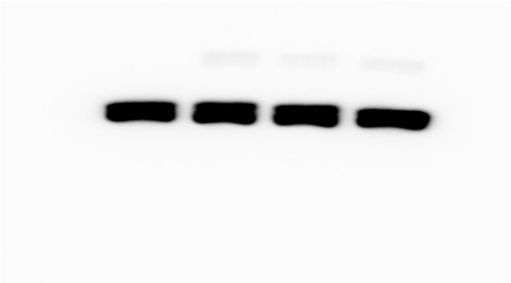

Figure 4h

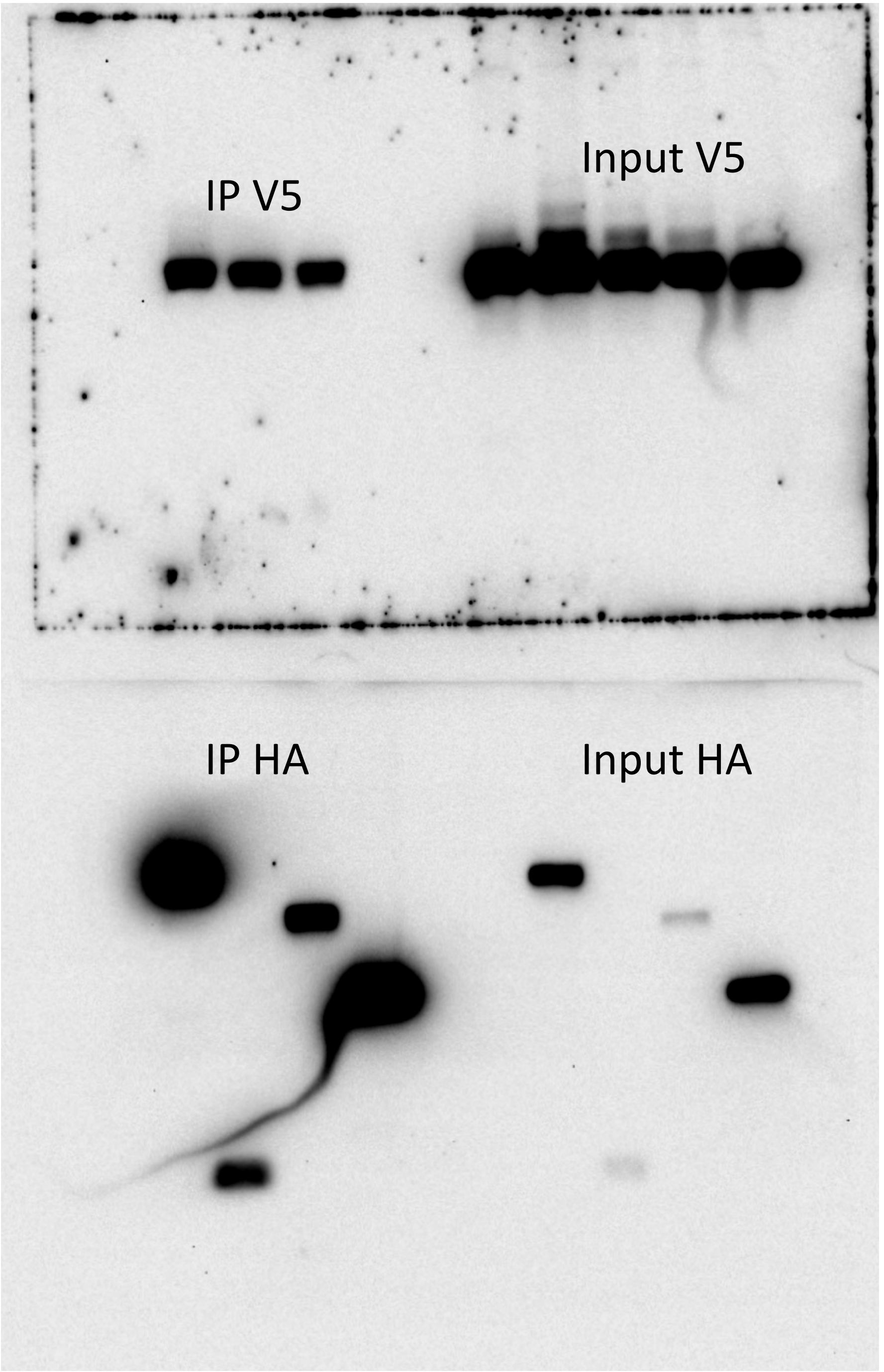

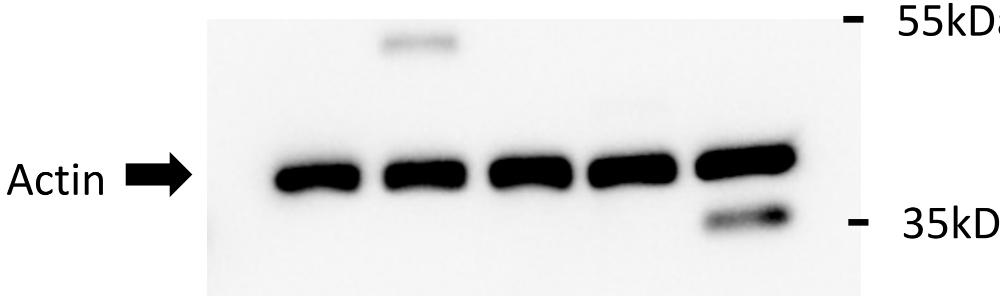

Figure 4i

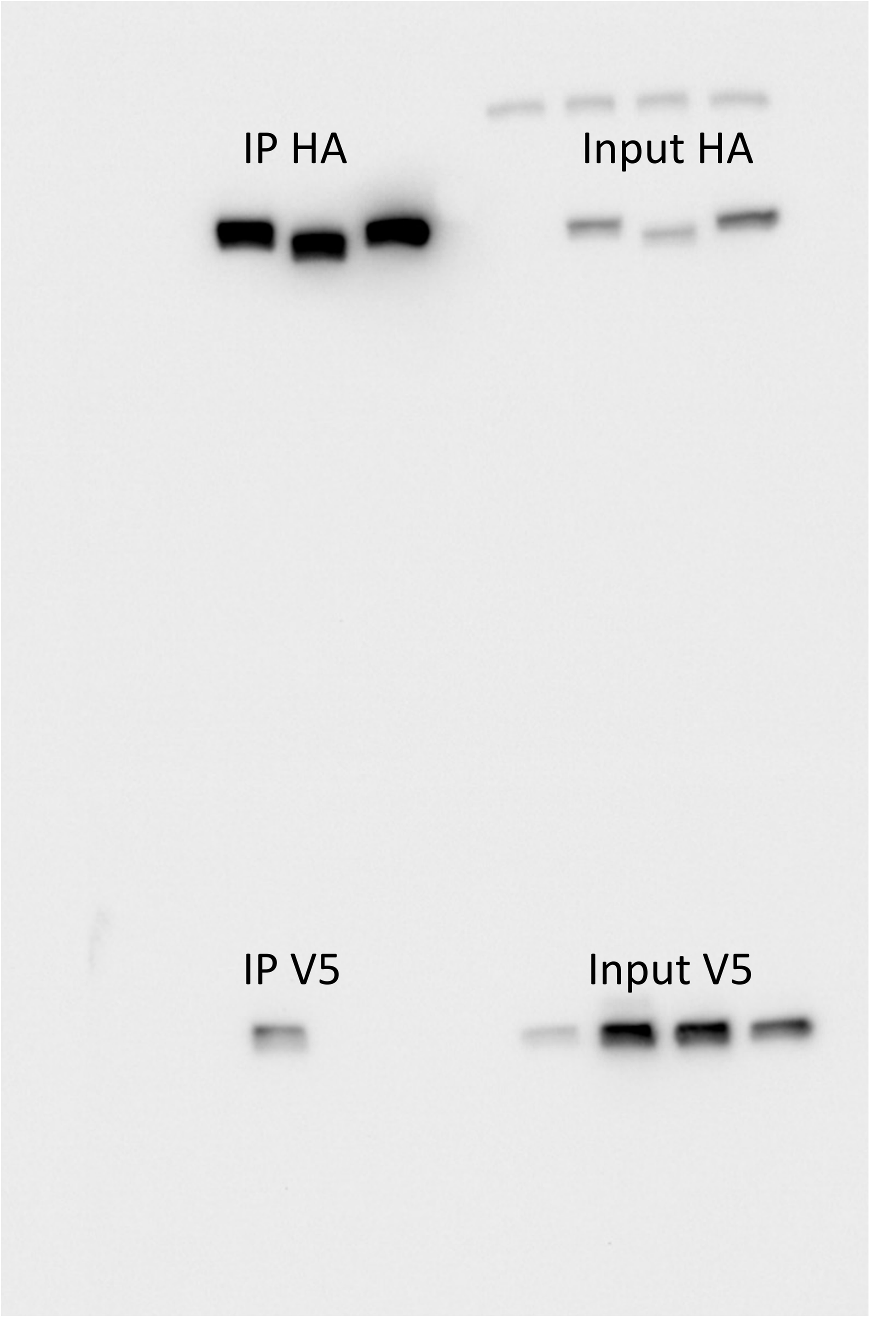

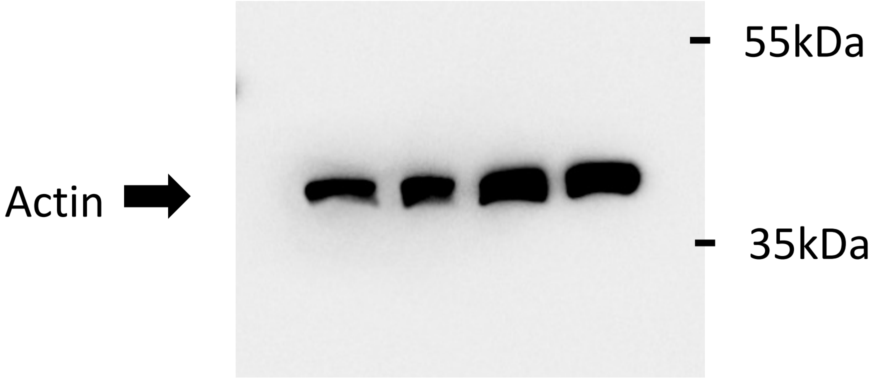

Figure 4j

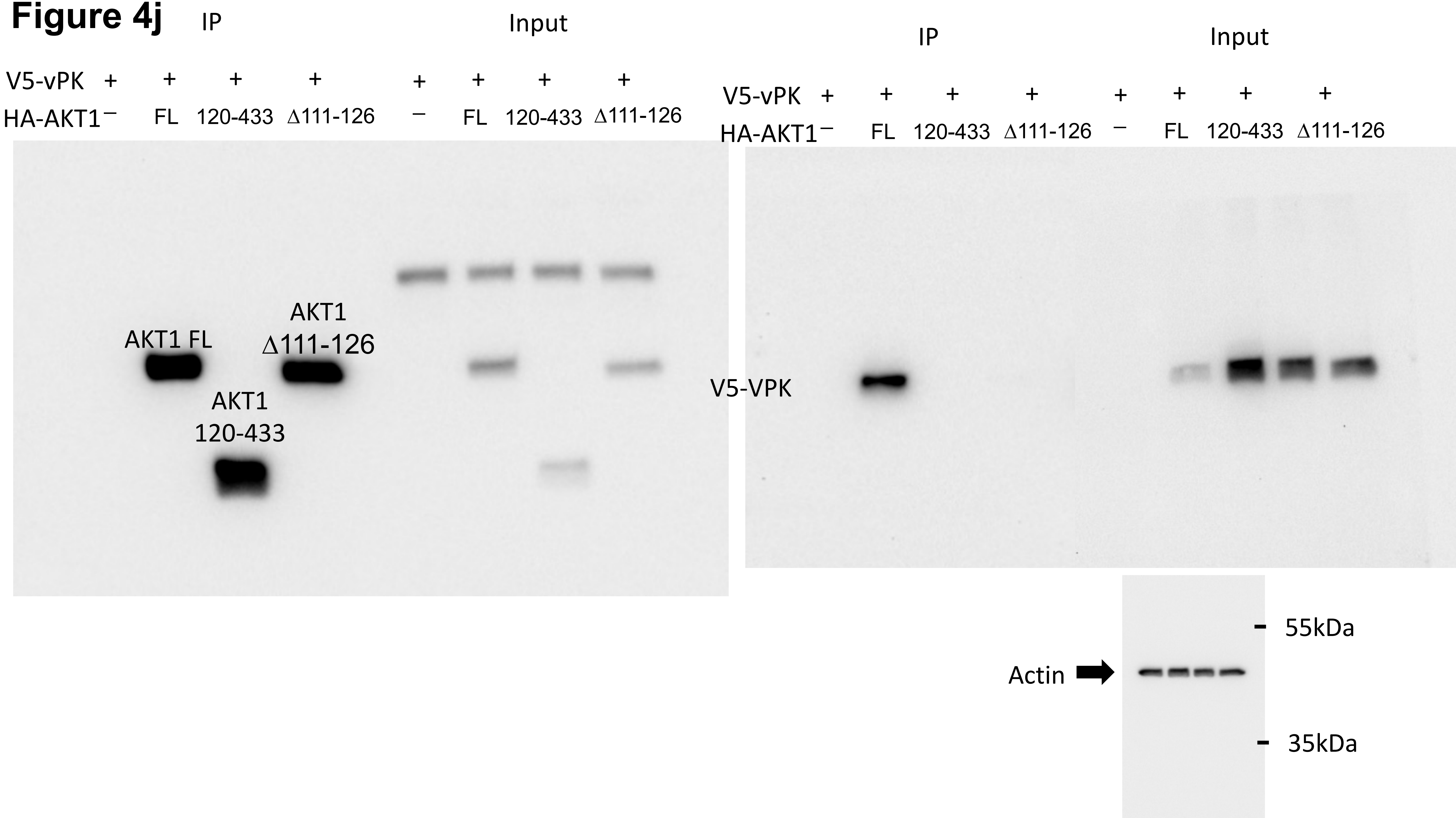
IP
Input
V5-vPK + + + + + + + +
HA-AKT1 – FL 120-433 Δ111-126 – FL 120-433 Δ111-126
AKT1 FL
AKT1 Δ111-126
AKT1 120-433
IP
Input
V5-vPK + + + + + + + +
HA-AKT1 – FL 120-433 Δ111-126 – FL 120-433 Δ111-126
V5-VPK
55kDa
Actin
35kDa

Figure 5a

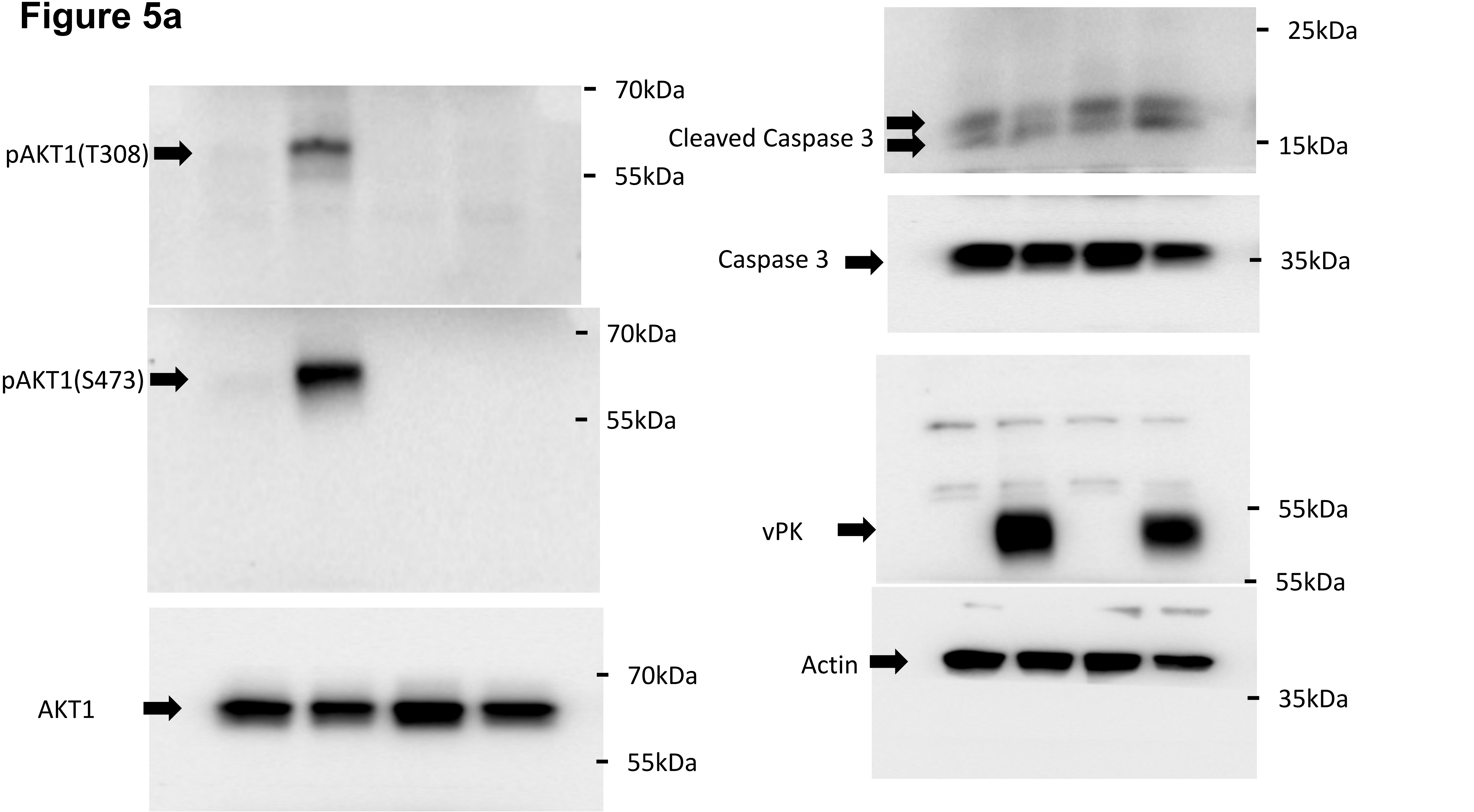

pAKT1(T308)
70kDa
55kDa
pAKT1(S473)
70kDa
55kDa
AKT1
70kDa
55kDa
25kDa
Cleaved Caspase 3
15kDa
Caspase 3
35kDa
vPK
55kDa
55kDa
Actin
35kDa

Figure 5h

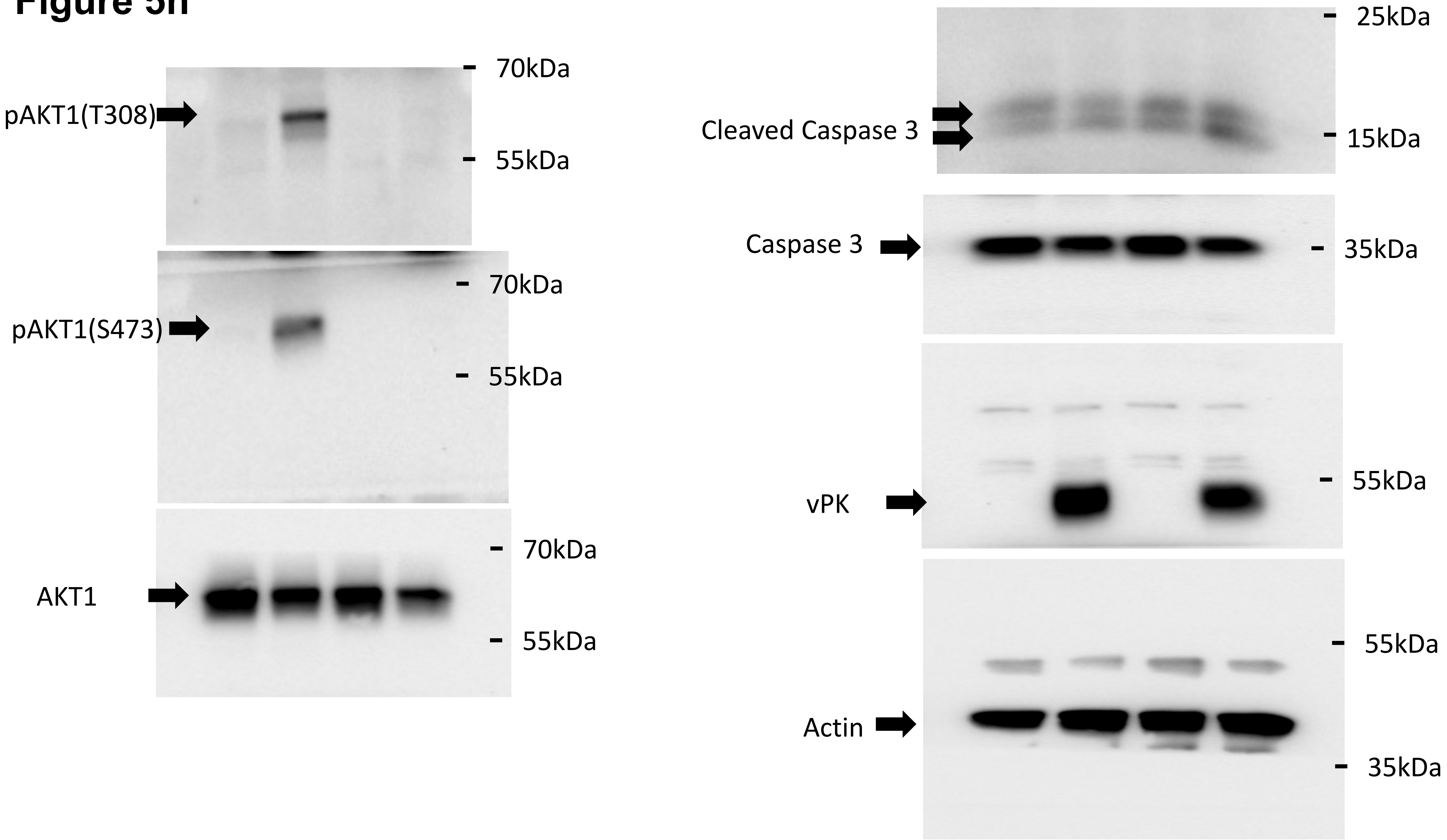

pAKT1(T308)
70kDa
55kDa
pAKT1(S473)
70kDa
55kDa
AKT1
70kDa
55kDa
Cleaved Caspase 3
25kDa
15kDa
Caspase 3
35kDa
vPK
55kDa
Actin
55kDa
35kDa

Figure 6d

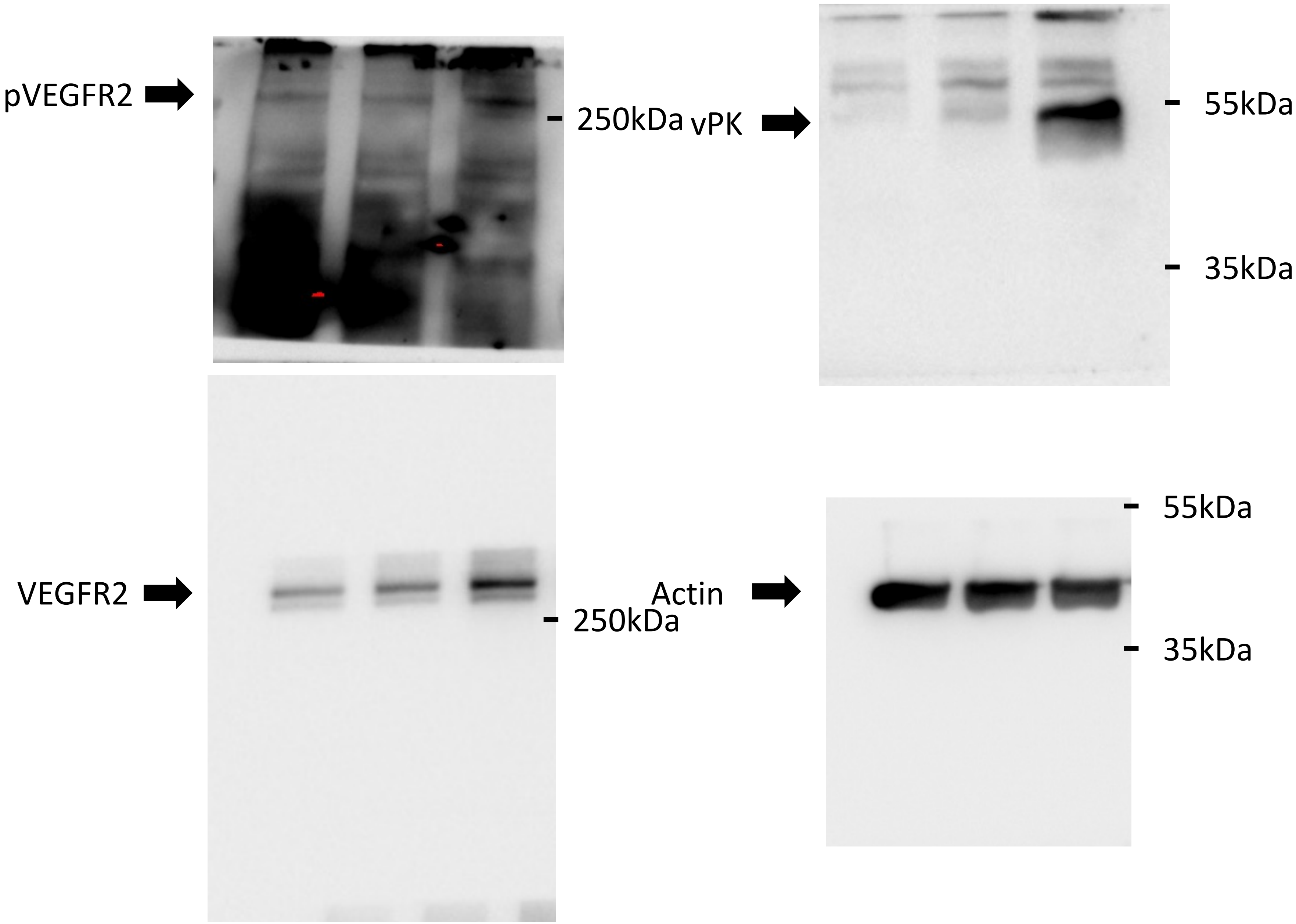
pVEGFR2
250kDa
vPK
55kDa
35kDa
VEGFR2
250kDa
Actin
55kDa
35kDa

Figure 6g

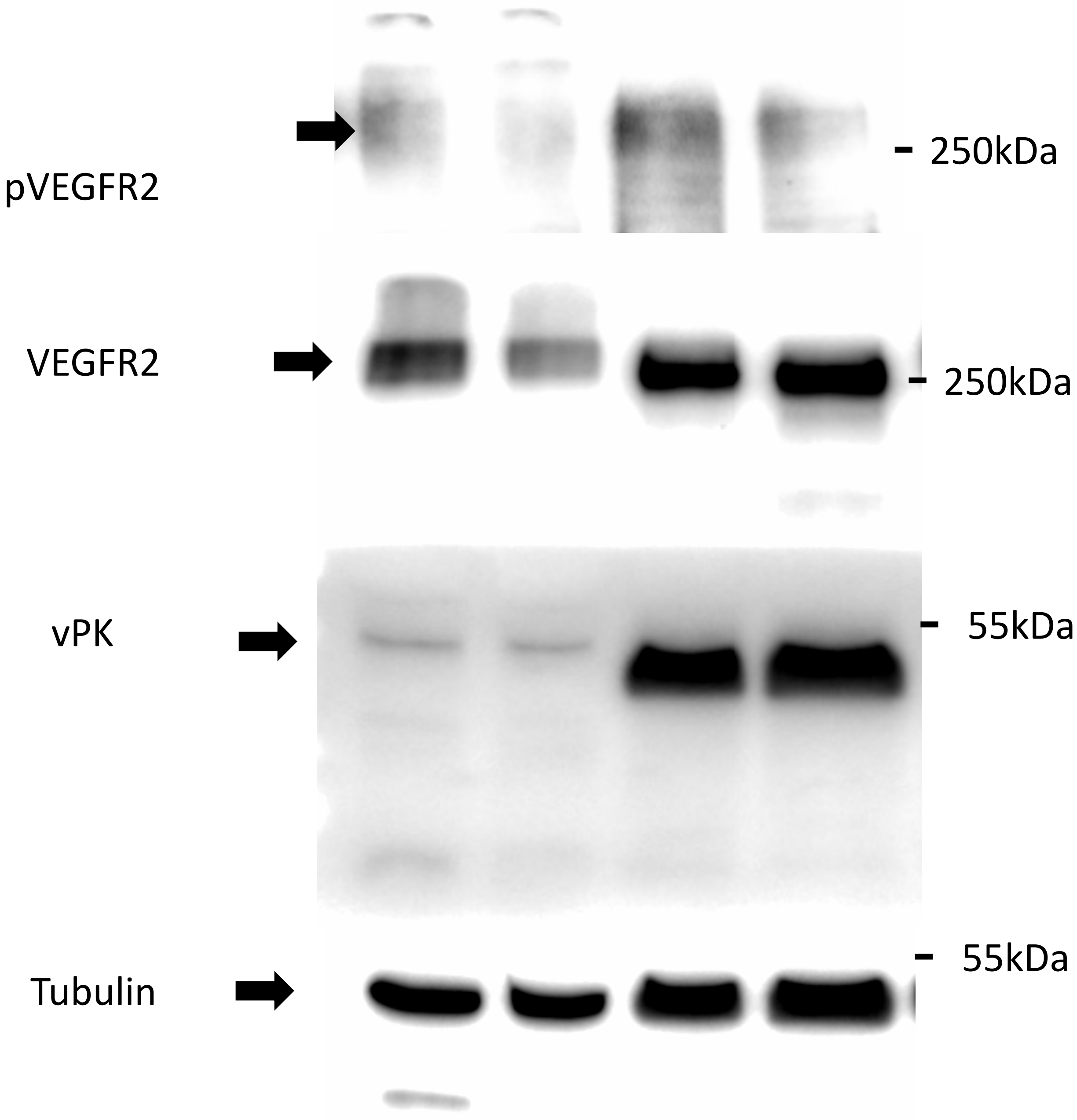

Figure 7d

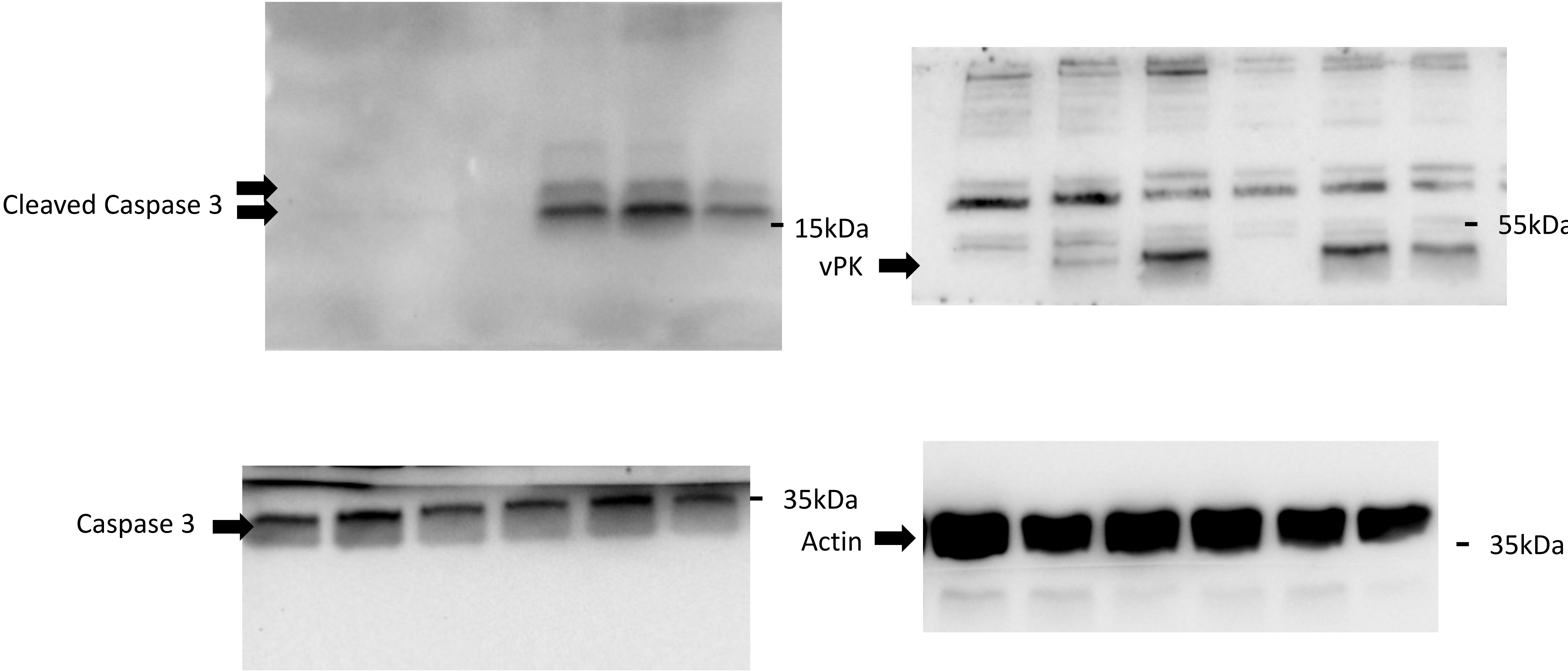

Cleaved Caspase 3
15kDa
vPK
55kDa
Caspase 3
35kDa
Actin
35kDa

Supplementary figure 1c

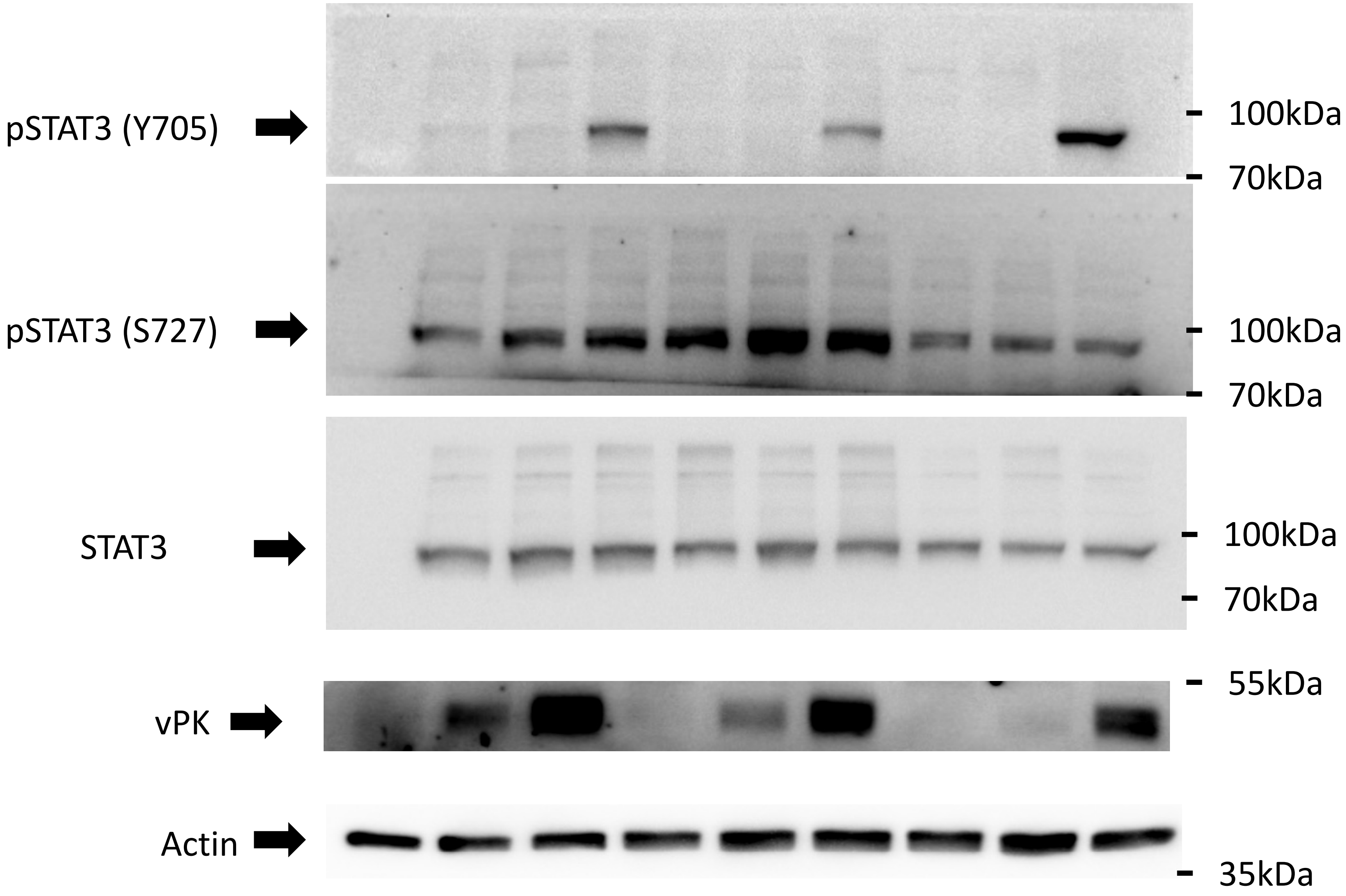

Supplementary Figure S1d

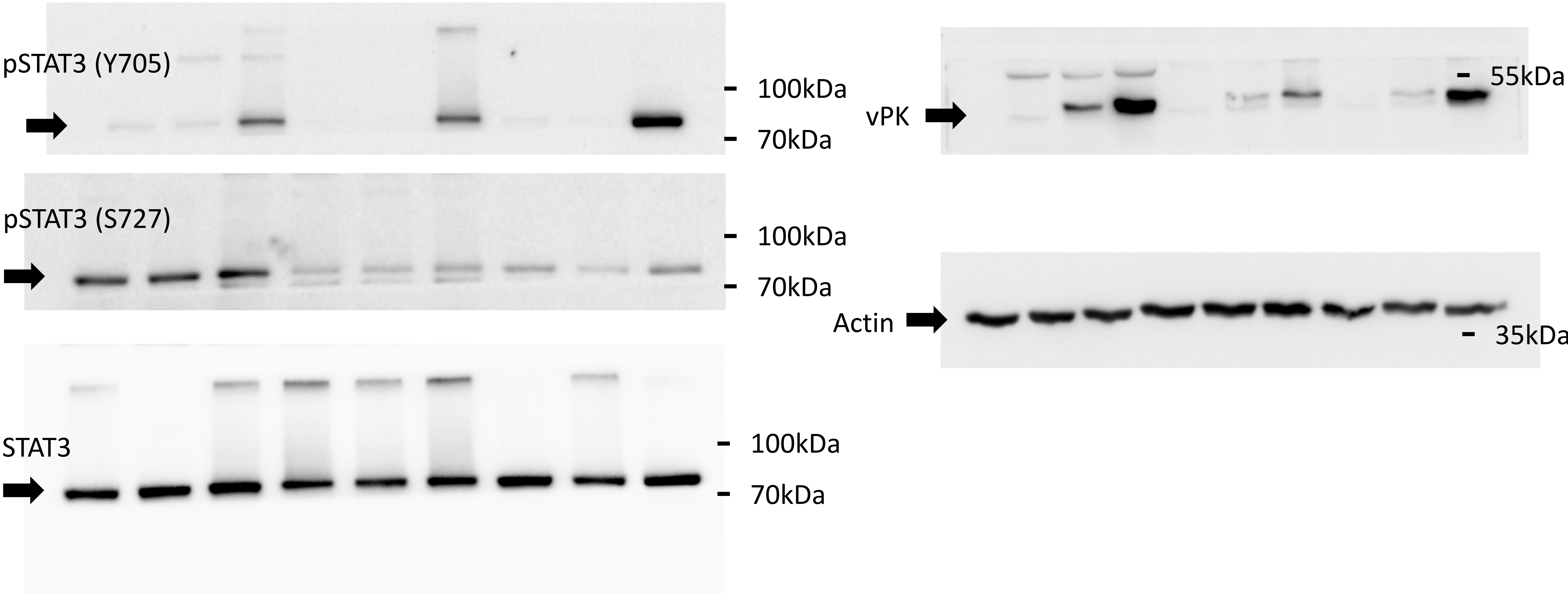

Supplementary Figure S2

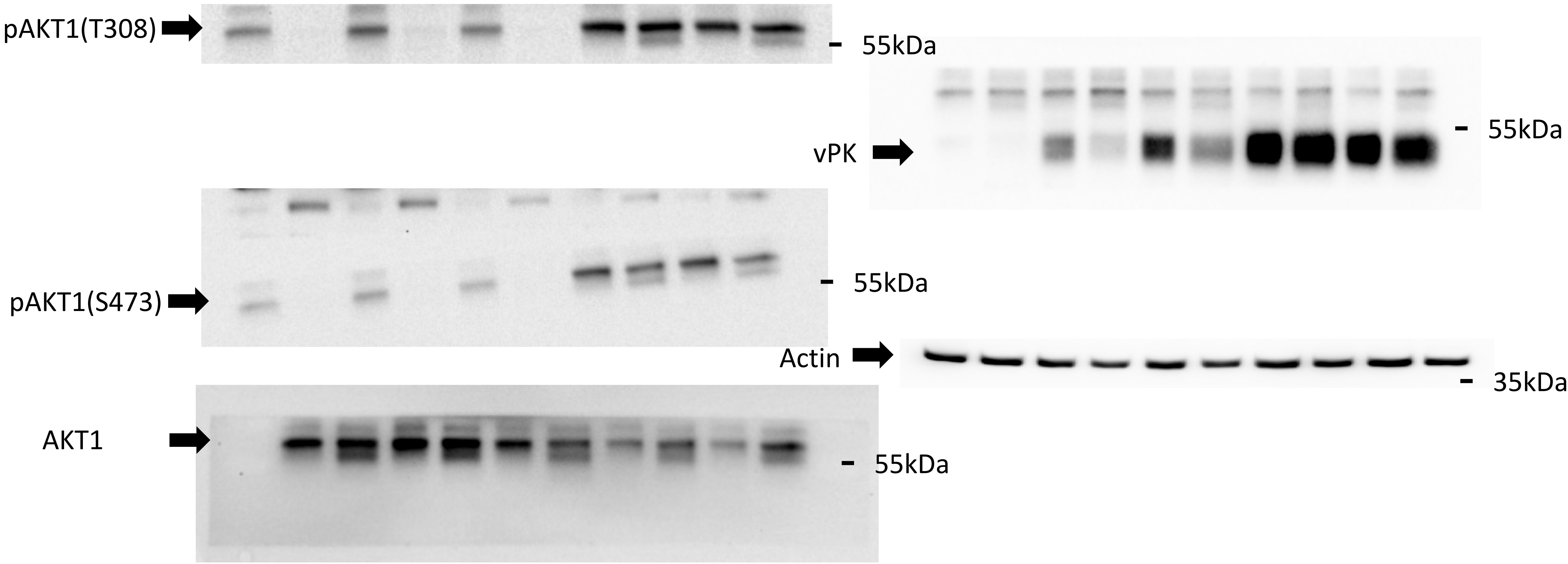

Supplementary Figure S3a and S3b

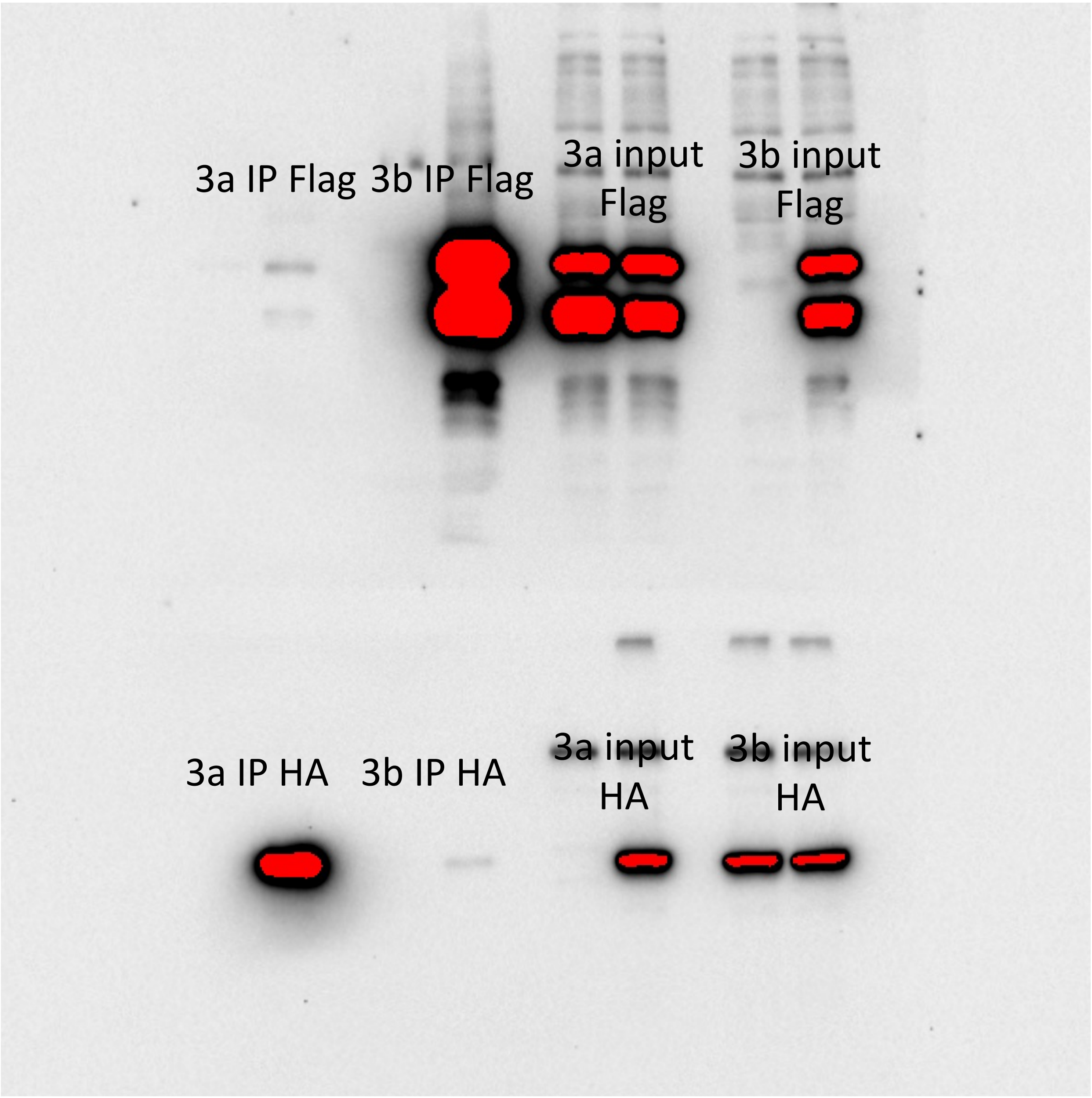

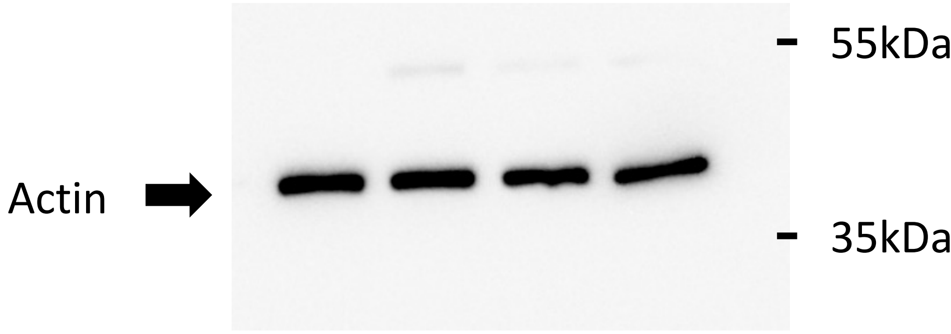

Supplementary Figure S5

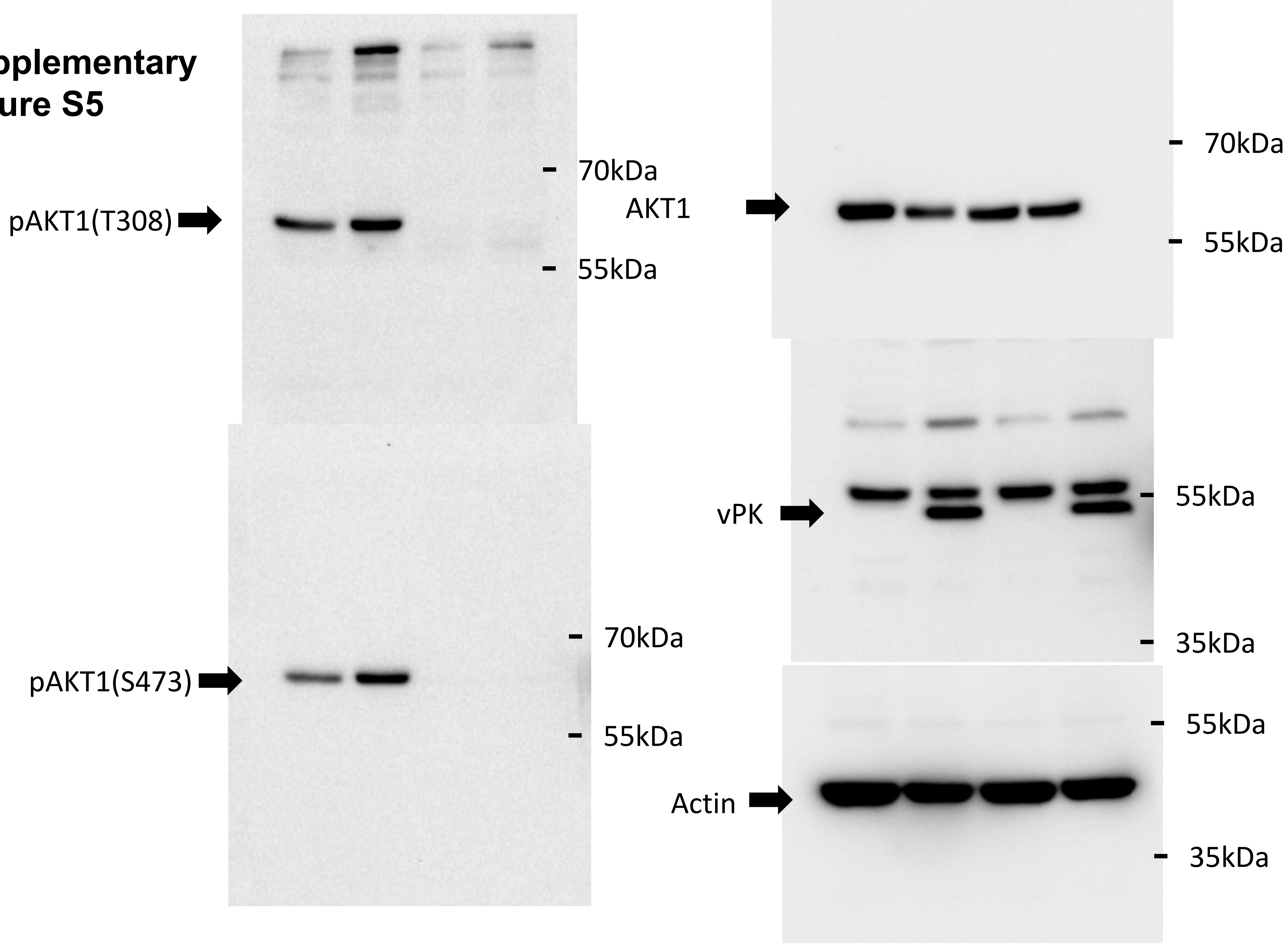

Supplement: Supplementary file 2 — Original Western Blots [file 41419_2023_6193_MOESM2_ESM.pdf]
